# Imperfect and askew: A review of asymmetric genitalia in araneomorph spiders (Araneae: Araneomorphae)

**DOI:** 10.1371/journal.pone.0220354

**Published:** 2020-06-15

**Authors:** Francisco Andres Rivera-Quiroz, Menno Schilthuizen, Booppa Petcharad, Jeremy A. Miller

**Affiliations:** 1 Biodiversity Discovery group, Naturalis Biodiversity Center, Leiden, The Netherlands; 2 Institute for Biology Leiden (IBL), Leiden University, Leiden, The Netherlands; 3 Endless Forms Group, Naturalis Biodiversity Center, Leiden, The Netherlands; 4 Faculty of Science and Technology, Thammasat University, Rangsit, Pathum Thani, Thailand; University of Western Ontario, CANADA

## Abstract

Bilateral asymmetry in the genitalia is a rare but widely dispersed phenomenon in the animal tree of life. In arthropods, occurrences vary greatly from one group to another and there seems to be no common explanation for all the independent origins. In spiders, genital asymmetry appears to be especially rare. Most known species show almost perfectly symmetrical genitals with the right and left sides being mirror images of each other. However, some examples of asymmetric genitalia have been studied and many other reports are scattered in the taxonomic literature. Based on a broad literature survey, we found several species in thirteen families with evidence of genital asymmetry, mostly expressed only in females. Our review suggests that spider genital asymmetries, although rare, are more common than previously thought and taxonomic descriptions and illustrations are a useful but not entirely reliable tool for studying them. Here we also report on directional asymmetry in the liocranid spider *Teutamus politus*, the first known case of morphologically asymmetric male genitals in Entelegynae spiders. Generalities, evolution and categorization of asymmetry in spiders are further discussed.

## Introduction

Genital asymmetry is a trait that has evolved independently several times in many animal groups. Invertebrates show a wide range of genital asymmetries with probably thousands of independent origins. Many, sometimes not mutually exclusive, explanations have been proposed, namely: i) morphological compensation for selected changes in mating position; ii) sexually antagonistic co-evolution; iii) cryptic female choice for asymmetric male genitalia; iv) different functions for the left and right side; v) one-sided reduction to save space and resources; vi) functional constraints: to function properly, the separate parts of the genitalia need to connect in an asymmetric fashion [1–4].

Asymmetries are often classified as fluctuating (FA), antisymmetry (AS) or directional (DA) [3, 5, 6]. This categorization is based on the degree and relative frequencies of the different chiral forms found in a population. FA describes slight asymmetric variation around a symmetrical mean; the appearance of this type of asymmetry is usually related to developmental instability [5, 7]. AS, also referred as ‘random asymmetry’ [8] describes cases where two mirror image forms, dextral and sinistral, are identifiable and within a population, occurring usually in equal or similar proportions [3]. Finally, DA refers to cases where only one asymmetric form is virtually always present [3]; this might be associated with mechanical, behavioral, or functional differentiation and selection of one asymmetrical form of the structures or organs [3, 9].

**Fig 1:**
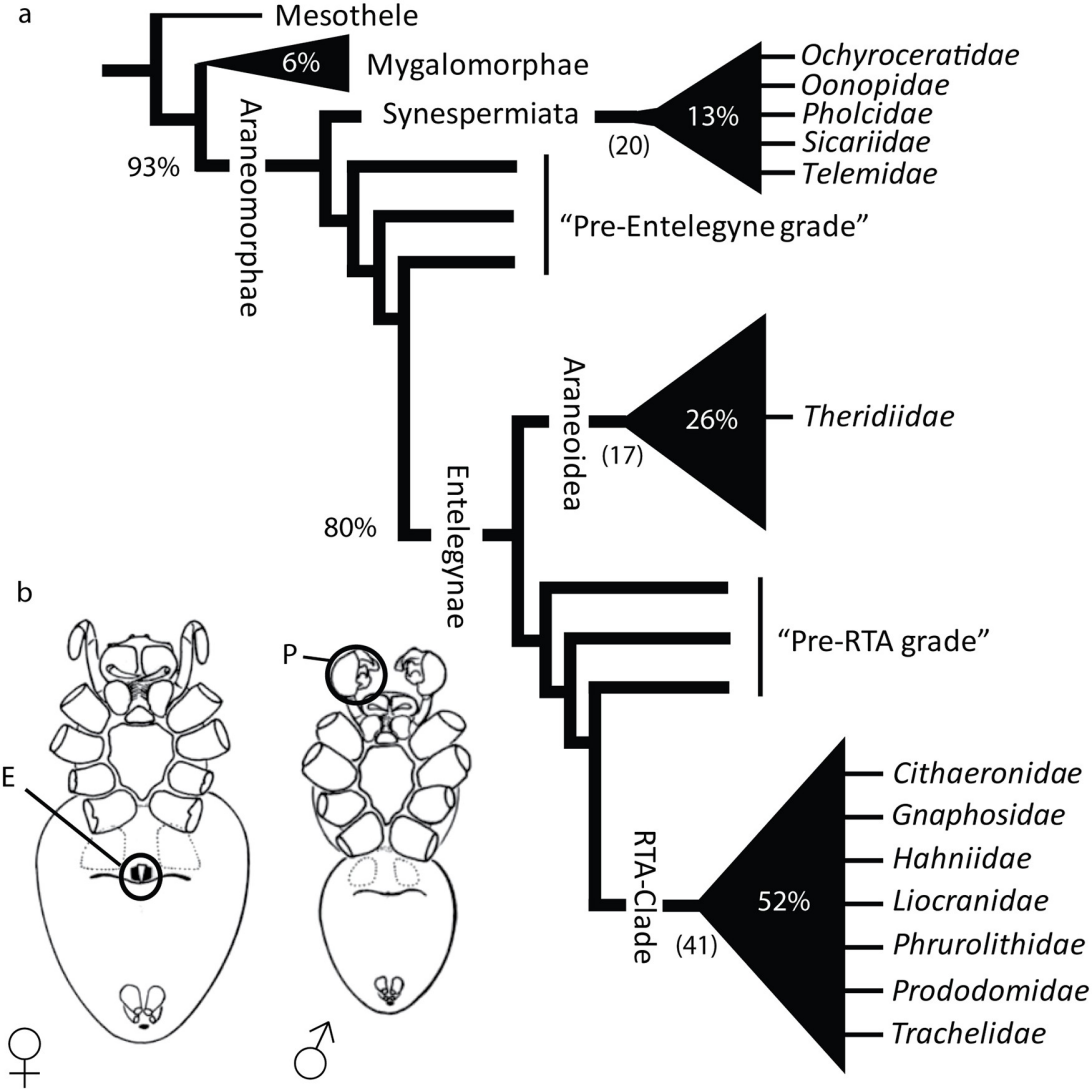
Spider relations and spider genitalia. a) Schematic tree based on a comprehensive spider phylogeny by Wheeler et al. [16]. Family names indicate where genital asymmetries have been observed. b) Ventral view of spider copulatory organs: ♀ Female genitalia or Epigynum (E) and ♂ Male Pedipalp (P); modified from Foelix [13]. Number of families per clade are indicated between parentheses. Proportion of species per clade in relation to the Order Araneae is also given.

Genital asymmetry, although rare as a whole, is a recurring phenomenon in a few groups of arthropods like mites, crustaceans, opiliones, and several insect orders. However, in spiders (Fig 1a), sexual asymmetries seem to be an exception [1–4, 10, 11]. In insects, copulatory mechanics and the presence of a single male genital structure located at the posterior end of the abdomen might explain the great incidence of genital asymmetry in this group [1, 3, 12]. In contrast, spiders have two male copulatory organs derived from a modified pair of leg-like appendages, here called pedipalps, that usually are matched to paired copulatory openings on the female genitalia, here called epigynum (Fig 1b) [13]. Pedialps are normally both used sequentially for sperm transfer during copulation and, in some cases, flexibility on the use of right and left sides has been observed [14, 15]. The paired nature of spider genital structures has been hypothesized to act as an “evolutionary buffer” to the development of genital asymmetry, especially on male genitals [1, 3, 11].

Asymmetries can be catalogued as genetic (larval) and environmental (post-larval) depending on the developmental stage where they are originated [6, 8]. In spiders, genital development is only apparent after the last molt. Therefore, the exact moment and mechanism by which asymmetry develops is difficult to interpret. Most cases of asymmetry in spiders have not been studied in detail or even discussed, with the notable exception of pholcids and theridiids [1, 3]. Nevertheless, taxonomic illustrations and descriptions reveal asymmetrical genitalia in other families. Genital asymmetry has been documented in male pedipalps (with variation in shape, size or even presence of the copulatory organs) and female epigyna. Female genital asymmetry can be further divided into external (position of copulatory openings) and internal (position and shape of sperm conducting and storing structures).

**Fig 2:**
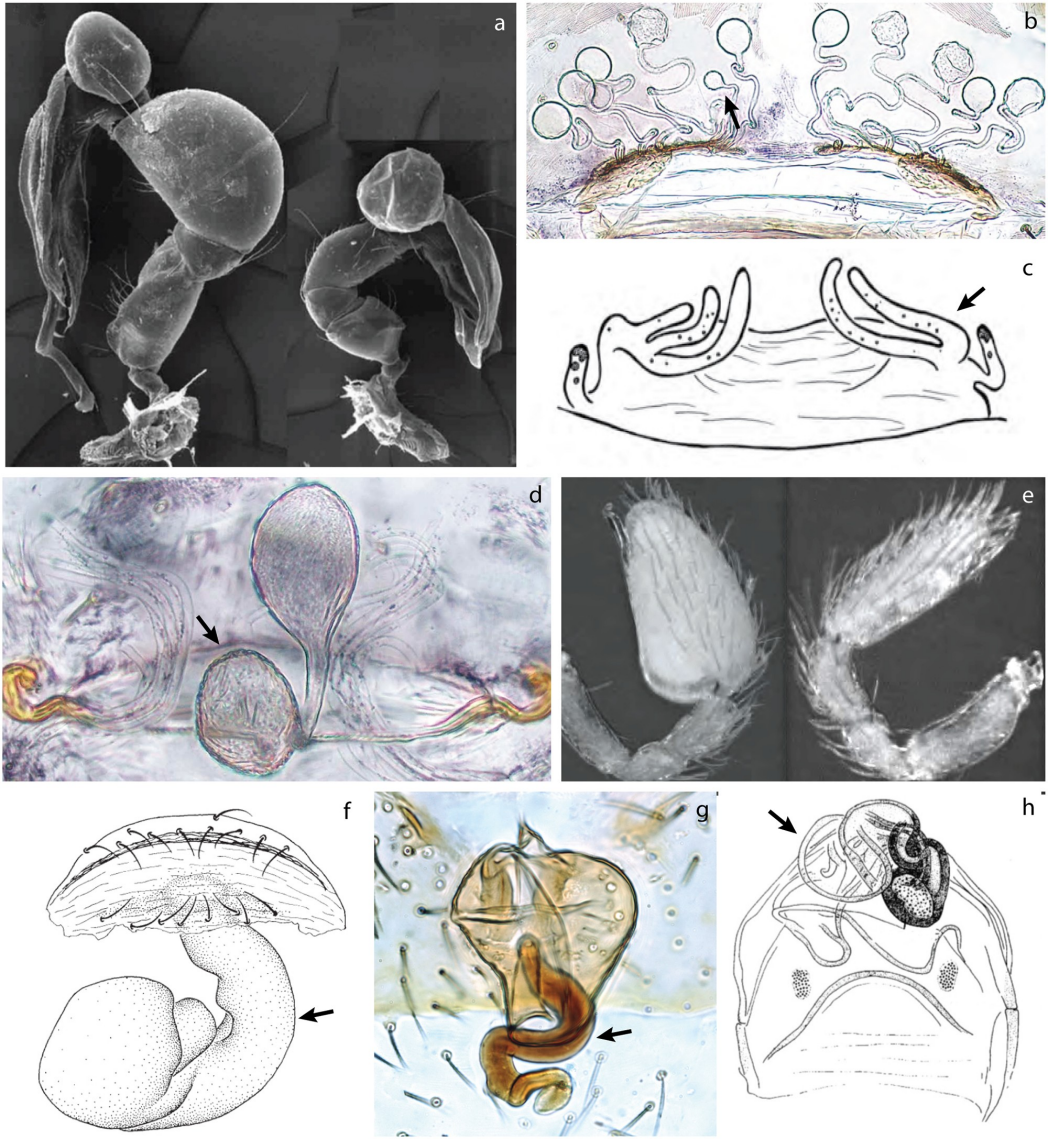
Examples of genital asymmetry in Synspermiata. a, e) male pedipalps, lateral view. b–d, f–h) vulva, dorsal view. a) Pholcidae: *Metagonia mariquitarensis*; modified from Huber [9]. b) Ochyroceratidae: *Althepus naphongensis*; modified from Li *et al*. [18]. c) Sicariidae: *Hexophthalma albospinosa*; modified from Magalhaes and Brescovit [19]. d) Ochyroceratidae: *Speocera cattien*; modified from Tong, *et al*. [20]. e) Oonopidae: *Paradysderina righty*; modified from Platnick and Dupérré [21]. f) Telemidae: *Telema exiloculata*; modified from Lin and Li [22]. g) Oonopidae: *Triaeris stenaspis*. h) Pholcidae: *Metagonia delicata*; modified from Huber [23]. Arrows indicate the asymmetric structure: b, c: number and development of spermathecae. d: size of spermathecae. f–g: direction of seminal receptacle.

**Fig 3:**
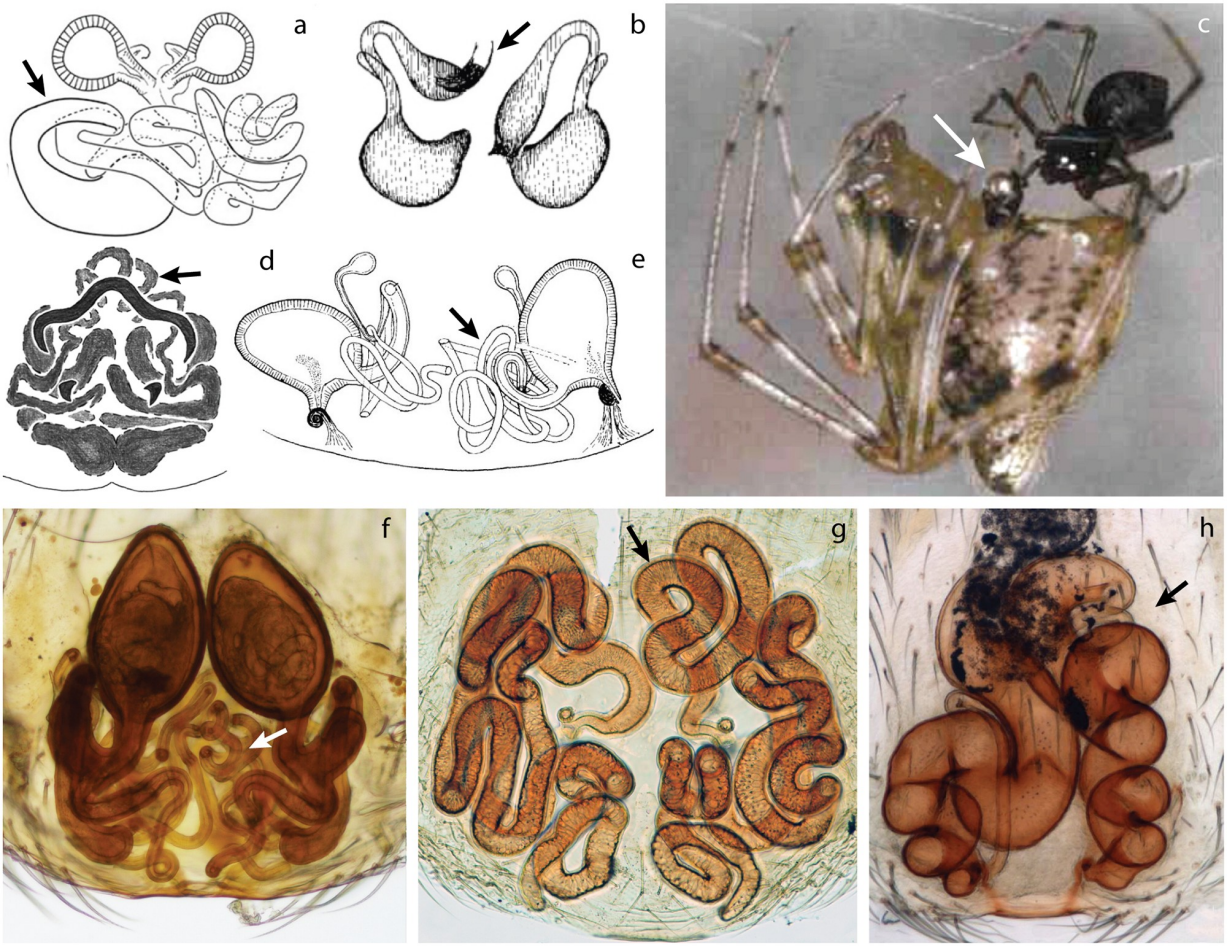
Examples of genital asymmetry in Entelegynae. a, b, d–h) vulva, dorsal view. c) male and female during copulation. a) Theridiidae: *Asygyna coddingtoni*; modified from Agnarsson [24]. b) Phrurolithidae: *Scotinella fratella*; modified from Dondale and Redner [25]. c) Theridiidae: *Tidarren sisyphoides*. [26]. d) Gnaphosidae: *Apopyllus gandarella*; modified from Azevedo *et al*. [27]. e) Hahniidae: *Iberina difficilis*; modified from Harm, 1966 [28]. f) Trachelidae: *Trachelas ductonuda*; modified from Rivera-Quiroz and Alvarez-Padilla [29]. g) Liocranidae: *Jacaena mihun*. h) Cithaeronidae: *Cithaeron praedonius*; modified from Ruiz and Bonaldo [30]. Arrows indicate the asymmetric structure: a, d–h: copulatory ducts. b: copulatory openings. c: male pedipalp.

Several independent origins in the spider tree of life have been found. However, all known cases have been reported in two major clades: Synspermiata and Entelegynae that include about 13% and 80% of known spider diversity, respectively (Fig 1a). Morphologically, Synspermiata spiders tend to have structurally simpler genitalia than entelegyne spiders in both sexes. Asymmetries in Synspermiata have been documented in two families: Pholcidae (Fig 2a and 2h) and Oonopidae (Fig 2e and 2g). Additionally, taxonomic descriptions and illustrations of some Ochyroceratidae (Fig 2b and 2d), Telemidae (Fig 2f) and Sicariidae (Fig 2c) depict female genital asymmetry too. In Entelegynae, examples appear more scattered and have been documented in two clades: Araneoidea and the RTA (*sensu* Wheeler et al. [16]). In the former, all known cases are found in the family Theridiidae (Fig 3a–3c) and in the latter, several examples have been illustrated in at least six families (Fig 3b, 3d–3h). Explanations for genital asymmetry in spiders are diverse and could include individual variation, natural selection, or sexual selection [1, 3, 11, 15, 17].

**Fig 4:**
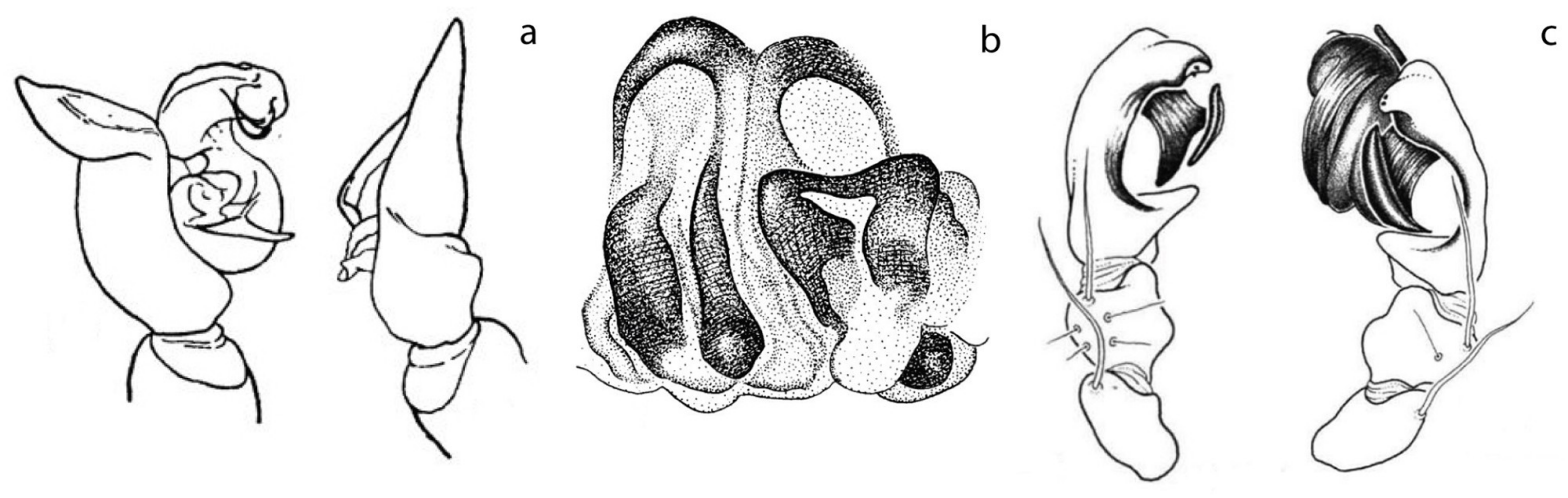
Examples of genital malformation in spiders. a, c) male pedipalps, posterior-lateral view. b) vulva, ventral view. a) *Lycosa ammophila*; modified from Kaston [42]. b) *Pardosa sagei*; modified from Kaston [42]. c) *Pimoa petita*; modified from Hormiga [40].

Spider genital asymmetry can be classified as follows: Fluctuating asymmetry (FA) is probably the most common type and has been documented in some Lycosidae [31–37], Pholcidae [38], and Oxyopidae [11, 39]. Other examples of seemingly asymmetric structures like the pedipalps of the one known male specimen of *Pimoa petita* [40] or the numerous documented anomalies and deformities [41–44] might easily be explained by developmental malformations (Fig 4).

Antisymmetry (AS) is the second most common form of asymmetry in spiders and has been documented in three genera of the Theridiidae (*Asygyna*, *Echinotheridion*, and *Tidarren*) (Fig 3a and 3c) [24, 45, 46]; one genus of Pholcidae (*Metagonia*) (Fig 2a and 2h) [23]; one genus of Phrurolithidae (*Scotinella*) (Fig 3b) [47] and scattered cases such as in Trachelidae (Fig 3f) [29, 48, 49], Cithaeronidae (Fig 3h) [50] and other RTA families. Directional asymmetry (DA) is the rarest type and, until now, it had only been reported in the pholcid *Metagonia mariguitarensis* (Fig 2h) [9]; DA has also been implied in some descriptions within the Oonopidae (Fig 2e) [21, 51], and in the liocranid *Teutamus politus* female genitalia [52]. All of these, other isolated reports, and scattered descriptions and illustrations, suggest that genital asymmetries in spiders have originated independently several times and their study might give better insights into how and when this phenomenon has evolved and the selective mechanisms behind it.

**Fig 5:**
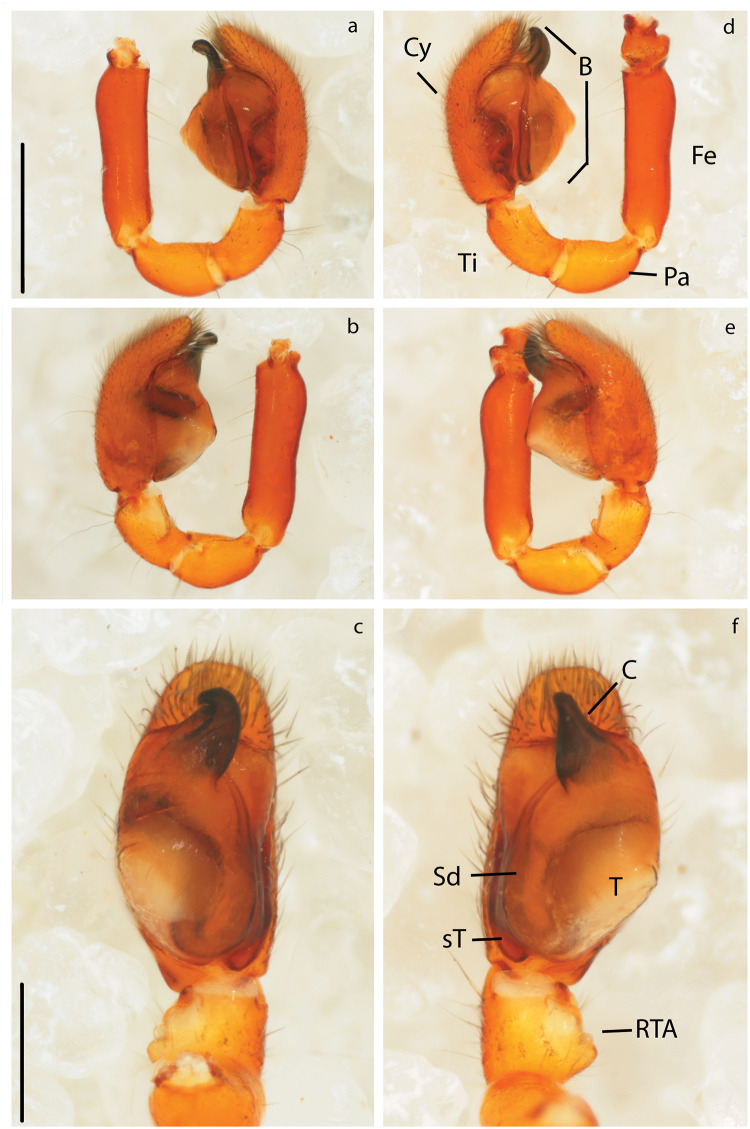
Male genitalia of *Teutamus politus* (unexpanded). Right pedipalp: a) prolateral view. b) retrolateral view. c) ventral view. Left pedipalp: d) prolateral view. e) retrolateral view. f) ventral view. Scale bars: **a**, **b**, **d**, **e** = 0.5 mm. **c**, **f** = 0.25 mm.

**Fig 6:**
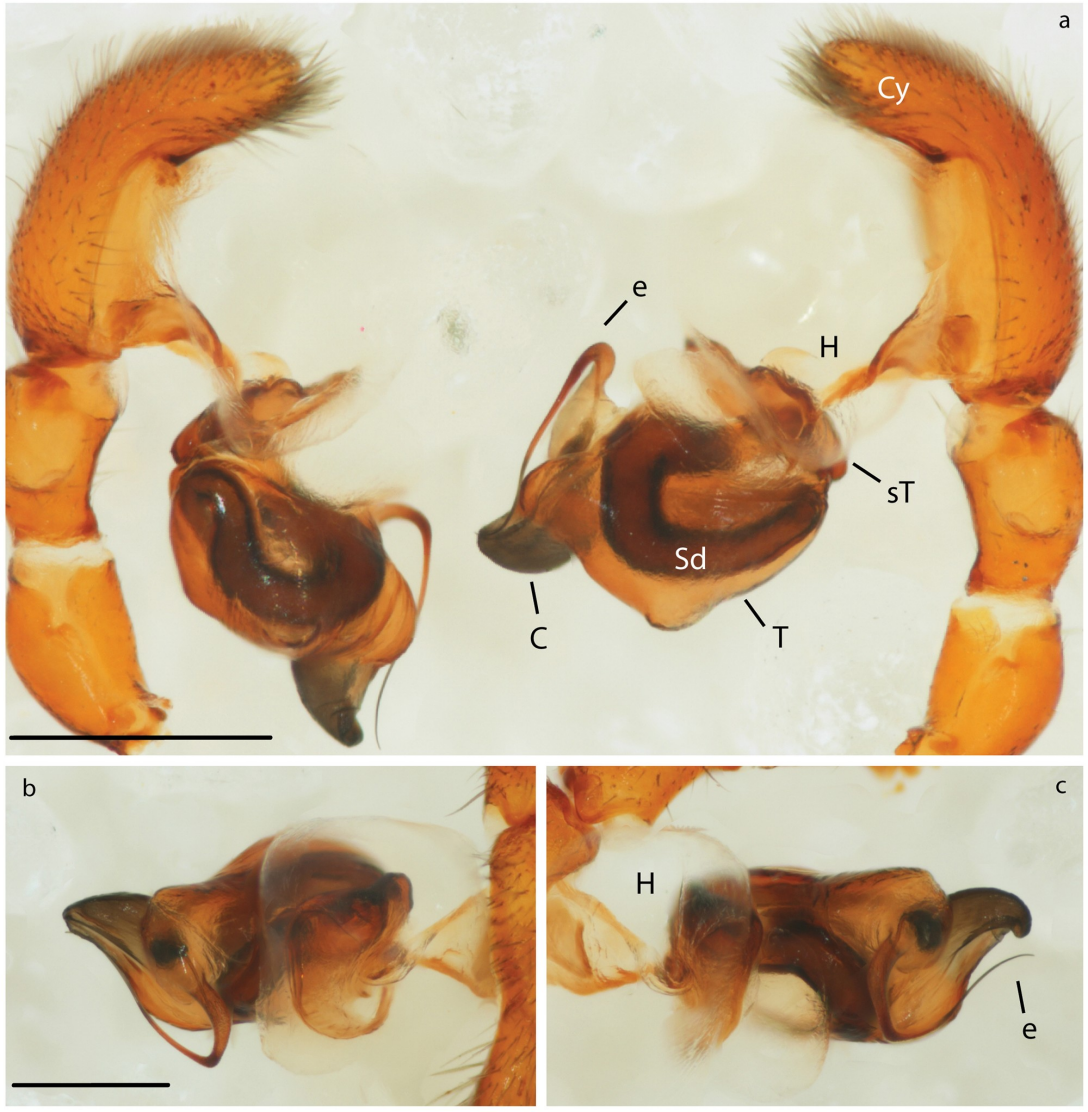
Male genitalia of *Teutamus politus* (expanded). a) comparative retrolateral view. b) left pedipalp prolateral view. c) right pedipalp prolateral view. Scale bars: **a** = 0.5 mm. **b**, **c** = 0.25 mm.

**Fig 7:**
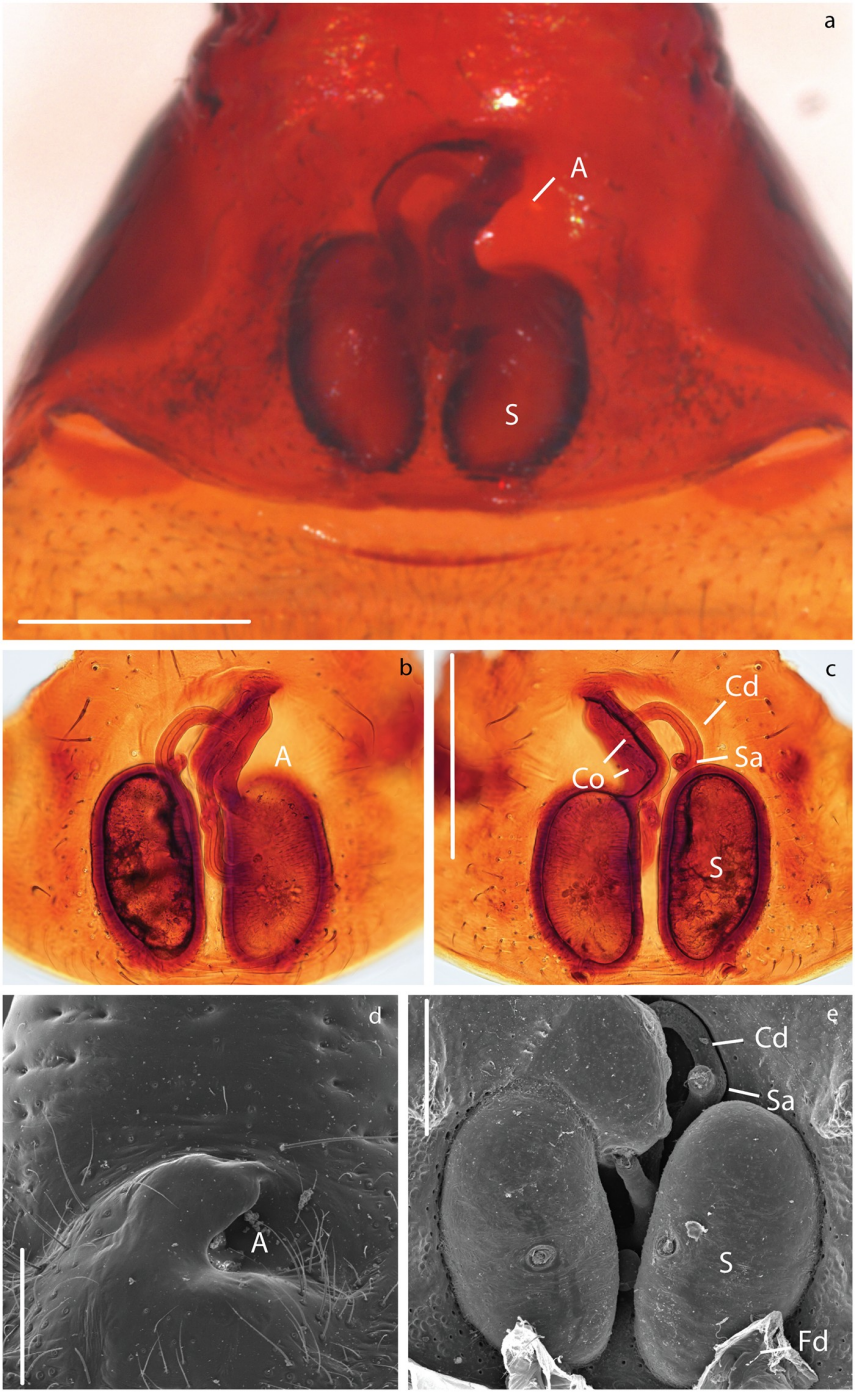
Female genitalia of *Teutamus politus*. a) epigynum ventral view. b) dissected and cleared vulva ventral view. c) same, dorsal view. d) vulva, ventral view, SEM. e) same, dorsal view. Scale bars: **a**, **b**, **c** = 0.25 mm. **d** = 150 um. **e** = 100 um.

A particularly interesting example is the Liocranidae where two different types of asymmetry are present [52–54]. For example, *Jacaena mihun* (Fig 3g) shows no external chirality, but internally the asymmetric copulation ducts are highly variable among individuals. Another example, *Teutamus politus* (Figs 5–7), shows external asymmetry in the female genitalia with both copulatory openings fused together in one atrium placed on the left side of the epigyne (see Deeleman-Reinhold [52]: fig 800, 801). Deeleman-Reinhold [52] mentioned female asymmetry as a diagnostic character for this species and noted that in all six of the specimens available for examination, the atrium is located in the left side. A revision of the genus *Teutamus* [53] also included external asymmetry in the female genitalia as a diagnostic character for *T*. *politus*, and expanded the sample of specimens examined; asymmetry in male pedipalp was not reported in either of these studies.

Here we present a general review of genital asymmetries in the spider literature, grouping them in previously described categories of genital asymmetry and discussing the existence of a new category of female genital asymmetry (here called Chaotic Asymmetry). We also analyzed the specific case of the species *Teutamus politus* by collecting new specimens in Thailand and documenting male and female genitalia using diverse morphological methods. This gives evidence of the first cases of both directional asymmetry in males and females, and developmental male genital asymmetry in Entelegynae spiders.

## Material and methods

### Literature review

We performed an informal search in taxonomic literature of several Synspermiata and Entelegyne families. Selection of publications was initially based on reported cases in literature [1, 3, 9, 11, 12] and then expanded depending on the occurrences found within each family. We did not contemplate cases of FA but this type of asymmetry is included in our discussion. We considered *T*. *politus* as a good model for testing basic hypotheses on genital asymmetry because of the clear external and internal morphology of female genitalia and Deeleman-Reinhold’s [52] note suggesting this could be a case of DA. Furthermore, we hypothesized that morphological or behavioral compensation for female genital asymmetry could be found in the male.

We considered male asymmetry as those cases that result in clear morphological differences between right and left pedipalp regardless of having a developmental or behavioral origin. Based on this, we also considered the pedipalp amputation that males of *Echinoitheridion* and *Tidarren* perform on themselves in our review; especially since the asymmetry has clear adaptive and evolutionary implications [15, 46, 55–57].

### Fieldwork

We selected study sites and collecting dates based on the relative numbers of collected adult specimens of *T*. *politus* mentioned in the literature [52, 53]. Fieldwork was carried out in Thailand between July 29^th^ and August 12^th^ 2018; here we sampled 12 sites in total: eight in Phuket Island (8°1.673’N 98°22.019’E, 144m; 8°1.816’N 98°22.375’E, 215m; 8°2.310’N 98°23.407’E, 135m; 8°2.353’N 98°23.365’E, 173m; 7°53.355’N 98°26.083’E, 132m; 7°53.384’N 98°26.102’E, 104m; 7°53.169’N 98°26.108’E, 88m; 7°53.409’N 98°26.067’E, 117m) and four more in Krabi Province (8°29.536’N 98°44.353’E, 93m; 8°29.572’N 98°44.367’E, 85m; 8°29.655’N 98°44.001’E, 60m; 8°29.592’N 98°43.907’E, 56m). We attempted to cover a variety of vegetation types ranging from relatively well preserved mixed forests to rubber and oil palm plantations. In each site we processed leaf litter using Winkler extractors and direct collecting on ground, among leaf litter and under rocks and logs. Hand collected specimens were kept alive in individual tubes. Winkler specimens were collected in a mixture of propylene glycol and 96% ethanol. All *Teutamus* specimens used in this study were collected under permit 5830802 emitted by the Department of National Parks, Wildlife and Plant Conservation, Thailand. Specimens were deposited in the collection of the Naturalis Biodiversity Center, Leiden, The Netherlands (RMNH.5084632–RMNH.5084651), and the Natural History Museum of the National Science Museum, Thailand (THNHM-I-12251–12252).

### Behavioral observations

Live specimens were kept individually in clean 15ml Falcon tubes and fed with termites every two days. Seventeen males and 19 females were selected and assigned unique numbers. Couples were formed preferably with specimens from the same locality. Spiders were placed in a Petri dish (diameter 5 cm, height 1 cm); each dish was divided by a paper wall with a small opening so spiders could roam freely but flee in case of aggression. Each couple was kept in the dish under constant observation for a period of about three hours. Travel logistics and specimen sensitivity (especially of males) to environmental changes, did not allow to further test different times and conditions. After observations, all specimens were sacrificed and stored in 96% ethanol.

### Morphological methods

Somatic characters and male sexual structures were photographed using a Leica MI6SC Stereomicroscope equipped with a Nikon DS-Ri2 camera. Female genitalia were dissected, digested using a pancreatine solution [58], cleared with methyl salicylate. Observations were made using semi-permanent slide preparations [59] in a Leica DM 2500 microscope with the same camera as above. Male genitals were expanded using 10% KOH and distilled water in three 3 min. cycles leaving the pedipalps in distilled water overnight to stabilize them for photography (modified from Shear [60]). Female epigyna and male pedipalps were prepared for SEM and mounted following Alvarez-Padilla and Hormiga [58] SEM images were obtained using a JEOL JSM-6480LV electron microscope.

The following abbreviations are used in the text and figures: **Female genitalia**: A, atrium; Cd, copulatory ducts; Co, copulatory openings; Fd, fertilization ducts; Sa, secretory ampullae (*sensu* Dankittipakul, *et al*. [53]); S, spermatheca. **Male genitalia**: B, male pedipalp bulb; Cy, cymbium; C, pedipalp conductor; E, embolus; Fe, femur; H, basal hematodocha; Pa, patella; RTA, tibia retro lateral apophysis; Sd, sperm duct; sT, sub tegulum; T, tegulum; Ti, tibia.

## Results

### Literature review

We reviewed publications that directly focus on genital asymmetry as well as taxonomic literature that allusively describe or illustrate asymmetrical morphology. We found more than 150 species across thirteen spider families with indications of asymmetric genitalia (Table 1) representing less than 0.3% of all spider species World Spider Catalog (WSC) [61]; and about 13.5% of all the currently valid species in the genera reviewed for this study. Synspermiata has at least five families (Ochyroceratidae, Oonopidae, Pholcidae, Sicariidae and Telemidae) where some kind of asymmetry has evolved accounting for ca. 90 species (Table 1). Asymmetry was found in both female and male genitalia; female asymmetry is more frequent, being found in at least five oonopid, three sicariid, two pholcid and two ochyroceratid genera. In addition, most genera in the Telemidae have evolved a single sac-like seminal receptacle; some species show seemingly asymmetric modifications of this sac, leaning and sometimes spiraling to one side (Fig 2; Fig 5 [62]; Fig 7a and 7b [63]). However, intraspecific variation has not been documented. Male asymmetry is less common, being found in three oonopid (*Aschnaoonops*, *Escaphiella*, and *Paradysderyna*) and two pholcid (*Metagonia* and *Panjange*) genera, and ambiguously suggested for two ochyroceratid species [20, 64]. Nevertheless, it is prevalent in *Escaphiella* and *Paradysderina*, where about 20 species show apparent directional asymmetry in male pedipalps (Fig 2e).

**Table 1:** Spider taxa with genital asymmetry reports in literature.

Family	Species	External (E) / Internal (I)	Female / Male	Type of asymmetry	Distribution	Source
**Synspermiata**						
**Oonopidae**	*Aschnaoonops marta*	E	M	AS/DA? ^1^	Neotropical	Platnick *et al*. [65]
	*Aschnaoonops meta*	I	F	AS? ^1,3^	Neotropical	“
	*Escaphiella* (8 spp)	E	M	AS/DA? ^1^	Neotropical	Platnick and Dupérré [51]*
	*Lionneta* (2 spp)	I	F	AS/FA? ^2,3^	Seychelles	Saaristo [66]
	*Ischnothyreus* (whole genus?)	I	F	AS/CA/FA?^3^	Tropical Asia	Edward and Harvey [67]; Tong *et al*. [68]; Brescovit *et al*. [69]
	*Paradysderina* (10 spp)	E	M, F	AS/DA? ^1^	Neotropical	Platnick and Dupérré [21]*
	*Reductoonops* (2 spp)	I	F	AS? ^1^	Neotropical	Platnick and Berniker [70]
	*Triaeris* (5 spp)	I	F	DA? ^3^	Pantropical	Platnick *et al*. [71]
**Ochyroceratidae**	*Althepus* (5 spp)	I	F	AS/FA? ^1,2,3^	South-East Asia	Deeleman-Reinhold [72]; Li *et al*. [18]
	*Speocera* (8spp)	I, E	M, F	AS? ^3^	Pantropical	Lin, *et al*. [22];Tong and Li [64]; Tong *et al*. [20]
**Pholcidae**	*Mesabolivar yuruani*	I	F	DA? ^1^	Venezuela	Huber [17]
	*Metagonia* (9 spp)	I	F	AS	South America	Huber [23]*;Ferreira *et al*. [73];Huber [74] Huber *et al*. [75]; Machado, Ferreira and Brescovit [76]; Perez-Gonzalez and Huber [77]
	*Metagonia mariguitarensis*	E male/ I female	M, F	DA	Brazil	Huber [9]*
	*Panjange lanthana* group (3 spp)	E	M	DA? ^1^	Philippines	Huber [11]
**Sicariidae**	*Hexophthalma* (3spp)	I	F	FA? ^3^	South America	Magalhaes, Brescovit, and Santos [19]
	*Loxosceles* (4spp)	I	F	FA	North to South America; Africa	Gertsch and Ennik [78]*; Lotz [79]
	*Sicarius* (14 spp)	I	F	FA	South America	Magalhaes, Brescovit, and Santos [19]*
**Telemidae**	*Cangoderses christae*	I	F	AS/FA? ^1,3^	Côte d’Ivoire	Wang and Li [80]
	*Kinku turumanya*	I	F	AS/FA? ^3^	Ecuador	Dupérré and Tapia [62]
	*Telema* (14 spp)	I	F	AS/FA? ^1,3^	East and South-East Asia	Wang and Li [63]; Wang and Li [81]; Lin and Li [82]; Lin, Pham and Li [22]
**Entelegynae**						
**Araneoidea**						
**Theridiidae**	*Asygyna* (2 spp)	E, I	F	AS	Madagascar	Agnarsson [24] *
	*Echinotheridion* (whole genus)	E	M	AS	Neotropical	Knoflach [46]*
	*Tidarren* (whole genus)	E	M	AS	America, Tropical Africa	Knoflach and van Harten [15]*
**RTA**						
**Cithaeronidae**	*Cithaeron* (2 spp)	I	F	CA	South America, South-East Asia, North Africa	Platnick [83]*; Platnick and Gajbe [50]; Ruiz and Bonaldo [30]
**Hahniidae**	*Neoantistea* (2 spp)	I	F	AS/CA/FA? ^1,2,3^	Nearctic	Opell and Beatty [84]
	*Hahnia* (3 spp)	I	F	AS/CA/FA?^3^	Palearctic	Harm [28]
	*Iberina mazarredoi*	I	F	AS/CA/FA?^3^	Spain	Ledoux [85]
	*Mastigusa* (2 spp)	I	F	CA? ^1,3^	Northern Europe	Almquist [86] Azarikina and Trilikauskas [87]
**Gnaphosidae**	*Apopyllus* (9 spp)	I	F	CA/FA? ^3^	Neotropical	Azevedo, *et al*. [27]
**Liocranidae**	*Jacaena mihun*	I	F	CA	Thailand,	Deeleman-Reinhold [52]; Dankittipakul, *et al*. [54]
	*Teutamus politus*	E, I	M^a^, F	DA	Thailand, Malaysia	Deeleman-Reinhold, [52]*; Dankittipakul, *et al*. [53] and this study*
	*Teutamus* (4 spp)	E, I	F	AS/FA? ^2^	Sumatra	Deeleman-Reinhold [52]*; Dankittipakul, *et al*. [53]
**Phrurolithidae**	*Scotinella* (2 spp)	E, I	F	AS	USA	Penniman [47]
**Prodidiomidae**	*Moreno ramirezi*	I	F	CA? ^3^	Argentina	Platnick, Shadab, and Sorkin [88]
**Trachelidae**	*Trachelas* (7 spp)	I	F	CA? ^1,2,3^	North and Central America	Platnick and Shadab [49]; Platnick and Shadab [48]; Rivera-Quiroz and Alvarez-Padilla [29]
**Lycosidae**	*Several cases of FA*, *here we list a few examples*:					
	*Delirosa karadagensis*	E	F	FA	Ukraine	Kovblyuk [36]
	*Geolycosa latyfrons*	E	F	FA	USA	Wallace [35]
	*Tsamanicosa subrufa*	E	F	FA	Australia	Framenau and Baher [37]

Summary of cases and types of spider genital asymmetry, mostly from taxonomic literature. (AS, antisymmetry; CA, chaotic asymmetry; DA, directional asymmetry; FA, fluctuating asymmetry). Tenuous asymmetry categorizationsindicated by: ?^1^ small sample sizes, ?^2^ imprecise illustrations, ?^3^ information ambiguous or incomplete.

* indicates where intraspecific variation is reported.

^a^ described in the present work.

In Entelegynae, more than 60 species in eight families show genital asymmetry. Almost half of the cases were found in the Theridiidae with ca. 35 species in three genera (*Asygyna*, *Echinotheridion*, and *Tidarren*). The rest are scattered among seven families in the RTA clade (Cithaeronidae, Hahniidae, Gnaphosidae, Liocranidae, Phrurolithidae, Prodidiomidae, and Trachelidae) (Table 1). Most genital asymmetry reports in Entelegynae include only female genitalia. From these, internal asymmetry was the most common, showing a wide range of variation on spermathecae and copulatory ducts (Fig 3d–3h). In comparison, external asymmetry was not as usual being found only in *Asygyna* (Fig 3a), *Scotinella* (Fig 3b) and *Teutamus* (Fig 7a and 7d). Male genital asymmetry in Entelegynae had only been reported in the theridiids *Echinotheridion* and *Tidarren* (Fig 3c); these two genera exemplify a unique behavior that results in genital mutilation. Developmental asymmetry, rather than behaviorally induced, had never been described in Entelegynae literature before this work.

**Remarks on *Teutamus politus*** Thorell, 1890

(Figs 5–7, S1–S2).

A total of 60 female and 35 male specimens were collected in Thailand. The whole series of specimens were used for external female genitalia and male pedipalps observation and comparison. All the specimens showed the same direction in the genital asymmetry. Five females and five males had their genitals dissected and prepared for detailed examination. Additional images documenting external intra-specific genital variation using standard views of the genitalia can be found in the supporting information files (S1 and S2).

### Male genital morphology

All pedipalp segments with the exception of the bulb (B) seem to be completely symmetrical. Bulbs show consistent differences in the conductor (C) shape between the left and right pedipalps. The left C is conical and straight (Figs 5f and 6b), slightly pointing towards the cymbium (Cy) in a lateral view (Fig 5d and 5f); while the right C is flattened, hook-shaped (Fig 6c) and pointing away from the Cy (Fig 5a and 5c). Besides this, a more subtle asymmetry was found on the flatter ventral area of the left palp tegulum (T) (Fig 5f and 5e). There is no apparent difference in the length and shape of the emboli (E) or the spermatic ducts (Sd).

### Female genital morphology

Externally, the epigynal plate is flattened and fused to the ventral scutum (Fig 7a). Copulatory openings (Co) are placed close together, forming an atrium facing the left side of the venter and located anteriorly to the bean-shaped spermatheca (Fig 7a–7c). Left spermatheca is slightly shorter than right one (Fig 7c). Copulatory ducts (Cd) are equally long. Right Cd anterior to the right spermatheca, left Cd located in between both spermathecae (Fig 7c and 7e). Asymmetric attachment of Cd to spermathecae with the right being anterior to that of the left one (Fig 7b and 7c). Both Cd have secretory ampullae (Sa) close to their middle portion (Fig 7b and 7c). Fertilization ducts (Fd) are short and simple, originating from the posterior end of the spermatheca and pointing in the same direction (Fig 7e).

### Behavioral observations

A total of 25 different couples were tested. Initially couples were formed with males and females from the same collection site. Males were more difficult to keep alive than females with most males dying within three days of collection. Due to this, males and females from different sites were also coupled. There were no successful observations of either courtship or mating. Spiders preferred to explore the dish or stand still and, whenever they got too close, they usually avoided each other. In general, interactions between females and males were brief and non-aggressive. Four females laid egg sacs in the Falcon tubes.

## Discussion

### Literature review

**Fig 8:**
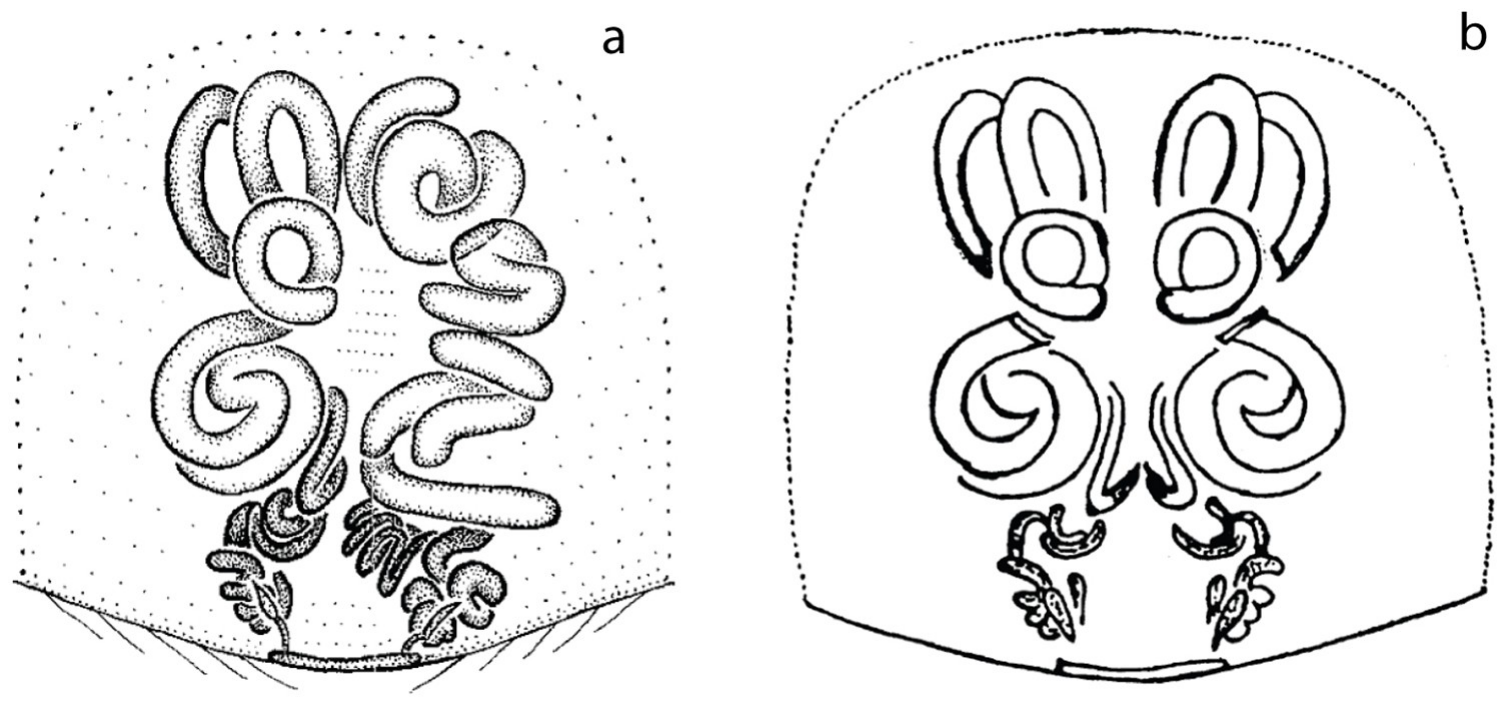
Example of illustration bias. Vulva, ventral view. a) *Cithaeron indicus*; modified Platnick and Gajbe [50]. b) Same; modified from Gajbe [89].

The taxonomic literature is the biggest repository of primary descriptive data on the world’s biodiversity. However, illustrations and description are difficult to interpret and might be influenced by the number of studied specimens, state of preservation, preparation artifacts and even illustration techniques. As an example, the species *Cithaeron indicus* shows clear asymmetric female genitalia in its original description [50] but appears symmetrical in a later publication [89] (Fig 8). Illustrators sometimes avoid introducing variation by drawing one half of a given structure and then tracing the other side based on it. This might simplify understanding and drawing some structures but could also lead to overlooking important information in the illustration process. Similar biases have been observed in some species of *Trachelas* [48, 49] and could be present elsewhere. As pointed out by Huber and Nuñeza [11], preparation artifacts might also play a role in the identification and interpretation of asymmetric structures. Weakly sclerotized internal genitalia (as that typically found in non-Entelegynae spiders) are often prone to create artifacts during specimen preparation and an interpretation without sufficient knowledge of intraspecific variation might be misleading. Entelegyne spiders tend to have more heavily sclerotized bodies being less prone to artifacts during the preparation process and allowing a more robust interpretation of their genital morphology.

Descriptions of male spider genitalia are also subject to preparation artifacts or methodological biases. Male genitalia preparation and examination is usually done by dissecting, studying and illustrating only one pedipalp. Although this is a very efficient approach and does not represent a problem on most occasions, some cases of asymmetric genitalia might go unnoticed. This has resulted in a more difficult assessment of male asymmetry; as an example, *Metagonia mariguitarensis* was considerd to be the only species with male genital asymmetry [9]. However, DA in males of *T*. *politus* had not been reported before, apparently because the right male pedipalp had been overlooked in previous descriptions. Similarly two *Speocera* species have their male pedipalps ambiguously described as “asymmetric” but no more details were given [20, 64].

In contrast, recent revisionary studies on the oonopid genera *Aschnaoonops*, *Escaphiella*, *Paradysderyna* and *Reductonoops* [21, 51, 65, 70] took special care in comparing the right and left male pedipalps revealing many more cases of genital asymmetry. In all of these, male pedipalps show consistent differences in bulb development and embolus shape between right and left (Fig 2e). In fact, for some species, enough specimens have been examined to confirm that asymmetry is directional [51].

Knowing intraspecific variation and having a big-enough specimen sample are crucial for confidently categorizing the types of asymmetry, and understanding the underlying evolutionary processes. Unfortunately, this kind of fine-grained data is rare (Table 2). A few studies have described intraspecific variation in detail. *Asygyna* [24], two species of *Scotinella* [47], *Tidarren cuneolatum* [15] several species of *Sicarius* [19] and the Pholcidae [9, 23] are some examples that report individual variation and (or) proportion of forms within the studied population. Other publications, like *Escaphiella* [51], *Paradysderina* [21] and *T*. *politus* [52] imply in the species descriptions that *all* the examined specimens show the same asymmetric morphology. Similarly, other studies deal with internal morphology variation by explicitly citing it in text as in *Cithaeron* [83]or showing it in comparative pictures as in *Jacaena* (Fig 8A–8D: Dankittipakul, *et al*. [54]) and *Loxoceles* (Fig 72–86:Getrsch [78]). Nevertheless, many other studies included in our revision show illustrations or photographs where asymmetric morphology is evident; but, no information about the variation within the species or proportion of forms is given.

**Table 2:** Number of specimens examined per species in literature.

Family	Species	Females	Males	Type of asymmetry	Source
**Synspermiata**					
**Oonopidae**					
	*Escaphiella gertschi*	446	285●	DA	Platnick and Dupérré [51]
	*E*. *itys*	529	220●	DA	“
	*E*. *tayrona*	27	28●	DA	“
	*E*. *betin*	17	18●	DA	“
	*E*. *acapulco*	3	1●	?	“
	*E*. *catemaco*	7	4●	DA?	“
	*E*. *chiapa*	52	32●	DA	“
	*E*. *Colima*	-	2●	DA?	“
	*Paradysderina asymmetrica*	4	7●	DA	Platnick and Dupérré [21]
	*P*. *boyaca*	1	2●	DA?	“
	*P*. *carrizal*	0	11●	DA	“
	*P*. *chinacota*	0	1●	DA?	“
	*P*. *fusiscuta*	2	1●	DA?	“
	*P*. *lefty*	2●	1●	DA?	“
	*P*. *monstrosa*	2	6●	DA	“
	*P*. *righty*	12	6●	DA	“
	*P*. *schizo*	0	1●	DA?	“
	*P*. *tambopata*	1	1●	DA?	“
**Pholcidae**	*Mesabolivar yuruani*	4●	1	DA?	Huber [17]
	*Metagonia delicate*	55 [6/7]●	34	AS	Huber [23]
	*M*. *uvita*	55 [22/32]●	32	AS	“
	*M*. *talamanca*	16 [5/9]●	7	AS	“
	*M*. *beni*	7●	3	?	Huber [74]
	*M*. *globulosa*	5●	2	AS	Ferreira *et al*. [73]
	*M*. *furcata*	1●	1	?	“
	*M*. *potiguar*	1●	1	?	“
	*M*. *diamantina*	1●	1	?	Machado, *et al*. [76]
	*M*. *mariguitarensis*	12●	4●	DA	Huber [9]
	*Panjange casaroro*	5	3●	DA?	Huber [11]
	*P*. *malagos*	4	1●	DA?	“
	*P*. *camiguin*	56	24●	DA	“
**Sicariidae**	*Sicarius thomisoides*	5●	5	FA	Magalhaes, Brescovit, and Santos [19]
	*S*. *fumosus*	5●	5	FA	“
	*S*. *crustosus*	5●	3	FA	“
	*S*. *lanuginosus*	3●	5	FA	“
	*S*. *yurensis*	5●	3	FA	“
	*S*. *peuensis*	5●	5	FA	“
	*S*. *gracilis*	5●	5	FA	“
	*S*. *boliviensis*	5●	5	FA	“
	*S*. *rupestris*	5●	7	FA	“
	*S*. *mapuche*	5●	6	FA	“
	*S*. *levii*	5●	6	FA	“
	*S*. *saci*	5●	5	FA	“
	*S*. *jequitinonha*	5●	2	FA	“
	*S*. *rugosus*	3●	3	FA	“
**Entelegynae**					
**Araneoidea**					
**Theridiidae**	*Asygyna coddingtonii*	15 [4/11] ●	5	AS	Agnarsson [24]
	*Asygyna huberi*	10 [2/8] ●	3	AS	“
**RTA**					
**Cithaeronidae**	*Cithaeron praedonius*	8●	4	CA	Platnick [83]
**Liocranidae**	*Jacaena mihun*	4●	6	CA	Deeleman-Reinhold [52]; Dankittipakul, *et al*. [54]
	*Teutamus politus*	113 (60)*●	67 (35)*●	DA	Deeleman-Reinhold [52]; Dankittipakul, *et al*. [53], and this study
**Phrurolithidae**	*Scotinella britcheri*	7 [4/3]●	N.S.	AS	Penniman [47]
	*S*. *fratellus*	24 [15/9]●	N.S.	AS	“

Details of the number of specimens examined per species and study. Species where asymmetry has been observed but number of specimens or variation are not mentioned in the original work are not noted here. (AS, antisymmetry; CA, chaotic asymmetry; DA, directional asymmetry; FA, fluctuating asymmetry). Individual variation indicated between brackets [right/left].

^●^ Indicates the asymmetric sex.

* Specimens examined in this study. N.S. not specified in the original study. *Echinotheridion* and *Tidarren* are not detailed here since all the valid species show the same type of asymmetry.

### Patterns of genital asymmetry

We found evidence of more than 150 cases of asymmetry in spider genitals in thirteen different families. Previous broad-scoped reviews noted only some examples in Pholcidae and Theridiidae [1–3, 11]. We identified multiple independent origins of asymmetry, some even occurring within the same family (as seen in Oonopidae, Pholcidae, Theridiidae Hahniidae and Liocranidae). Reports on insects suggest that genital asymmetry rarely appears isolated and is usually a shared trait between closely related species [3, 4, 90]. Here, we found some similar patterns with several species within a genus showing at least one type of genital asymmetry. Some cases like *Jacaena mihun* and *Teutamus politus* were seemingly isolated (until more cases are confirmed). However, we found some conspicuous examples of asymmetry shared between closely related species. These are the cases of male asymmetry in *Escaphiella* and *Paradysderina*, female asymmetry in *Asygyna*, *Metagonia* and *Trachelas*, and the emasculatory behavior in all the species of *Tidarren* and *Echinotheridion* (arguably closely related groups [46, 57, 91, 92]). This pattern is more common in the Synspermiata, but was also observed in Entelegynae (Table 1). Although the known number of cases and families with asymmetrical genitalia has increased significantly, this still represents less than 0.3% of all known spider species. The low incidence of genital asymmetry in spiders has been mainly explained by the presence of two sperm transfer structures in the male [1, 3]. Huber, *et al*. [1] remarks that in comparison to insects, most spider asymmetry originates in females instead of males. Many examples support this hypothesis, which also fits a cryptic female choice hypothesis [10]. Nevertheless, we found numerous “new” examples of male asymmetry hidden in the taxonomic literature (Table 1), highlighting the many cases in the Oonopidae where male asymmetry has apparently not coincided with modified female genitalia (further discussed in DA). Huber, *et al*. [1] also observed that most insect asymmetry evolves first as DA, while most (or all) spider asymmetry appears firstly as AS. Here we found that DA might not be as rare as previously thought. Examples of DA are included in Table 2 and discussed below. Many spider asymmetries seem to fit in the AS category, although only a handful have been evaluated for the appearance of right or left-sided asymmetries within a sample as in Phrurolithidae and Theridiidae [24]. Also, we found some cases in which female copulatory ducts are long, coiled and entangled in a way that does not fit any of the three known types of asymmetry. We called this chaotic asymmetry (CA) because the great variation between individuals of the same species does not allow distinguishing either a dextral or a sinistral form.

Other cases difficult to assess are: the reduction of spermathecae to a single receptacle, as seen in some oonopids [67, 69, 71, 93], pholcids [73, 76, 77], and telemids [22, 62, 63, 80–82] (Fig 2f and 2g); and the presence of odd numbered spermathecae in some sicariids [19, 78, 79, 94], ochyroceratids [18, 72, 95] (Fig 2b and 2c) and probably some mecymaucheniids [96]. Both phenomena can sometimes generate a seemingly asymmetric morphology. Although good illustrations and photographs of these are available in literature (e.g. Figs 20: Magalhaes, Brescovit, and Santos [19]; Figs 14 and 19: Li *et al*. [18]; Fig 8: Lin, Pham and Li [22]; Fig 7: Wang and Li [63]) only some cases in the Sicariidae [19, 78] have reported intraspecific variation.

As mentioned earlier, a correct interpretation of the type of asymmetry based only on the available literature is complicated. Intraspecific variation and proportion of forms are key pieces of information to distinguish the type of asymmetry and the evolutionary mechanisms behind it; however, these details are often overlooked. Here we include examples that, to the best of our knowledge, fit the definition of each type of genital asymmetry and give hypotheses that could explain their origin.

### Fluctuating Asymmetry (FA)

This kind of asymmetry is defined by van Valen [5] as “the inability of organisms to develop in precisely determined paths”. In other words, FA refers to small random morphological fluctuations around a symmetric mean [3, 5, 38, 97, 98]. FA incidence, relation to environmental factors, and its influence within populations has been studied on some Lycosidae and Pholcidae [17–21]. Here we found that some cases, like the hahniid *Neoanthistea*, some more lycosid, oonopid, and telemid genera (mentioned as FA? in Table 1), and other “malformed” specimens in literature might be cases of FA.

Similarly, the great intraspecific variation observed in the female genitalia of some sicariids [19, 78], range from asymmetries in number, size and shape of spermathecae to almost symmetric structures. This suggests that asymmetries in this family and similar cases in the ochyroceratid *Althepus* [18, 72] might be fluctuating. A few species that show AS (*Scotinella britcheri* and *S*. *fratella*) and all species with CA (*Cithaeron praedonius*, *Jacaena mihun*, among others) also have a range of morphological variation in female internal genitalia within the population. However, these variations are clearly bimodal (as seen in our examples of AS) or larger than the usual 1–2% observed in FA [7]. Thus we do not consider them to be fluctuating; these and other examples are discussed in the following sections.

### Antisymmetry (AS)

This kind of asymmetry describes cases where two mirror image forms, dextral and sinistral, are identifiable and within a population, usually occurring in similar proportions. Evidence from snails [99], crustaceans [6], and insects [6, 90, 100] suggest AS to be an evolutionarily unstable or transitional state between symmetry and DA [3, 6, 101]. In spiders, the co-ocurrence of these two kinds of asymmetry within the same genus has only been found in the pholcid genus *Metagonia* [9, 74].

Besides *Metagonia*, AS has been reported in at least two entelegyne families: Theridiidae and Phrurolithidae. Although the evolutionary scenario is different in each of the cases, it is interesting to observe the sex biased incidence of AS. In *Asygyna* and *Scotinella*, asymmetry has only been reported in females; while the theridiids *Echinotheridion* and *Tidarren* only show asymmetry of male pedipalps. Sex biased incidence of AS has also been observed some insect groups like Odonata, Orthopthera, Mantodea, and others [1, 2, 90].

Antisymmetry in female genitalia has been confirmed in three genera: *Asygyna* [24], *Metagonia* [23, 73, 74], and *Scotinella* [47]. All of these show both, dextral and sinistral forms within the studied samples. In *Scotinella*, two basic forms with some range of variation in-between were described. Nevertheless, no significant predominance of either within the studied populations was found [47]. The only known example of male AS is the one induced by an uncommon genital automutilation behavior. Notably, all known species in the theridiid genera *Echinotheridion* and *Tidarren* share this trait. In these genera, male spiders show no preference for either left or right pedipalp self-emasculation; furthermore, females show completely symmetric genitalia suggesting that no selection of right or left male forms is done by females. Experiments and observation on some species of *Tidarren* have shown that males can display two mating positions being able to inseminate any of the female spermathecae [1, 15]. This particular phenomenon has been related to other evolutionary oddities in these genera like mandatory mate consumption from females and extreme sex dimorphism in size [15, 46, 55–57, 91, 92, 102].

With the exception of the studies on male emasculation [15, 46, 55, 56, 102], the mechanical, behavioral or functional implications of AS have not been reported. Huber [17] suggest that AS in Pholcidae might respond to the exaggerated development of internal genital structures forcing a reduction in one of the sides, becoming asymmetrical. Agnarsson [24] explains the AS in *Asygyna* by sexual selection by female choice, either by reducing copulation times (leaving less chance for potential predators) or by discriminating males according to their abilities to introduce sperm. A similar scenario could also explain the case of *Scotinella*.

### Chaotic Asymmetry (CA)

This new category of asymmetry does not fit the definition of any of the three traditional types. In species of this type, females usually develop long and convoluted copulation ducts where the great variation between specimens does not allow a clear distinction between a dextral and sinistral form. All known examples of this type of asymmetry are found in the Entelegynae clade. Platnick [83] mentioned for *Cithaeron praedonius* (Cithaeronidae): “No two females show identical patterns of epigynal duct coiling; for that matter, no individual specimen shows identical coiling of the ducts of the right and left sides”. Similar morphological variation (Fig 3d–3h) has been observed in *Jacaena*, (Liocranidae) [54]. Apparent CA has also been observed in *Apopyllus* (Gnaphosidae) [27], *Neoantistea* and *Mastigusa* (Hahniidae) [84, 86, 87], *Moreno* (Prodidiomidae) [88], and *Trachelas* (Trachelidae) [29, 48, 49]. However, the variation within each species is not known, therefore, their categorization as CA is highly tenuous.

The origin of these internal genital modifications has not been investigated and its relation to a functional differentiation between sides or packing of other internal organs cannot be ruled out. We hypothesize that the development of this kind of asymmetry is related to complexity in internal female genitalia and this could explain the absence of examples in the genitally simple Synspermiata. The absence of a clear right/left pattern and great variation between individuals suggest that copulatory duct shape is not under a strong selection. This might be related to a simplification in pedipalp sclerite complexity and embolus length (as seen in *Trachelas*, *Jacaena* and *Moreno*). In contrast, some *Apopyllus* and *Mastigusa* males have fairly complex male genitals with an extremely long embolus that usually coils around the bulb. In the case of *Apopyllus*, female ducts show slight asymmetries between right and left sides and authors mention internal variation between conspecific females. This genus also shows intraspecific variation in the RTA and external genitalia and it is hypothesized to be an instance of male-female coevolution [27] which could be further explained by mechanical fit of genitalia during copulation in a female choice context *sensu* Eberhard [103] (see also the discussion on DA in *T*. *politus*).The cases of *Cithaeron indicus* [50], *Moreno ramirezi* [88] and both *Neoantistea* [84] species are doubtful; in the former, the male is not known, and in *Moreno* and *Neoantistea*, species were described based on just one female or variation was not documented. Therefore, the observed asymmetry could be fluctuating, antisymmetric, a developmental abnormality or even an artifact of preparation.

If pedipalp bulb sclerite reduction is related to the appearance of CA, the question would be why is it so rare? Within Entelegynae, several groups have reduced male pedipalp complexity; however, CA has not evolved nearly as many times. Long and convoluted ducts are hypothesized to be a way of avoiding premature fertilization and discriminating between different males sperm [104]. Although in many species male embolus deposits the sperm directly in the spermatheca (i. e. *Anyphaena accentuata* [105]), several cases have been found where female ducts are much longer than the male embolus (i. e. *Clubiona pallidula* [105]). In these cases, sperm transport by the female is necessary and pre- or copulatory stimulation may be related to it [104]. The “lengthening-hypothesis” in *Sparassidae* showed a correlation between emboli and copulatory duct length and complexity [106]. Also, this study show that in this family, evolution tended to be towards elongating instead of shortening. We could speculate that CA appears when long ducts are a preexisting condition, and its shape is not under selective pressure by male intromittent structures. Then, the shape of the ducts could vary randomly without compromising copulation but still keeping the sperm screening advantages predicted by the cryptic female choice model. More research on the physiological means of sperm transport and copulation mechanics of these species could shed some light on the evolution of CA.

### Directional Asymmetry (DA)

In insects, DA is the most common type of asymmetry [1, 2]; however, in spiders, DA seems to be quite rare. In Synspermiata literature, only the pholcid *Metagonia mariguitarensis* had been confirmed as DA, [9]. However, after our survey, we identified several reports of consistent one-sided asymmetries in other members of this clade. Some species of *Escaphiella* and *Paradysderina* show an extreme underdevelopment of the right palp in comparison to the left one [21, 51]. From these, *E*. *gertschi* and *P*. *carrizal*, among others (Table 2) had enough specimens checked to confirm directionality (more than 200 specimens reported for *E gertschi* and *E*. *itys*!). Other cases like *E*. *acapulco* or *P*. *boyaca*, had only a few specimens reported and were considered to be inconclusive (marked with “?” in Table 2). Other seemingly consistent male genital asymmetries have been described for three *Panjange* species of the *lanthana* group [11], *Aschnaoonops marta* [65], and at least six species of *Paradysderina* [21]. Likewise, female internal genitalia of *Mesabolivar yuruani* [17]; and some species of *Ischnothryeus* [67, 68, 93], *Paradysderina* [21], *Reductoonops* [70] and *Triaeris* [71] show asymmetries that seem to be consistent within their species. Nevertheless, either the number of specimens examined is low or variation within the species is not explicitly described making it difficult to confirm directionality.

The story seems to be different for Entelegynae spiders where more complex development of genitals might inhibit the evolution of directional asymmetry. Although implicit in the description of *Teutamus politus* female genitalia by Deeleman-Reinhold [52], the present study is the first report of DA in the entelegyne clade. *Teutamus politus* is also the first example of developmental male genital asymmetry in the Entelegynae. Previously, male asymmetry in this clade was only known from teratogenic specimens and the unique AS phenotype created by self-emasculation in *Tidarren* and *Echinotheridion*.

Putative cases of male DA in *Escaphiella* and other oonopids have only been observed in males and may not be related to modifications in female genitalia [51, 65]. In all these cases, underdevelopment of one pedipalp might indicate a functional specialization of one side over the other. Observations in other oonopids have shown that during copulation both palps are inserted simultaneously [107, 108]. In the cases we found, pedipalps asymmetry could potentially lead to a reduction in copulation times, a more efficient transfer of sperm or a better stimulation of the female genitalia. Similarly, female genital asymmetry in other oonopids like *Triaeris*, has not been linked to male pedipalp modifications. Oonopid internal female genitalia has proven to be one of the most complex in Synspermiata [93, 107–111]. More studies on it and on male-female interactions might lead to interesting discoveries like the sperm control mechanisms on [108, 109, 111] or the potential parthenogenesis in some *Triaeris* species parthenogenetic [71].

In contrast, to the cases before, directional genital asymmetries in *M*. *mariguitarensis* and *T*. *politus* have been found in both sexes, which might indicate that selection by female choice is the underlying cause. In *T*. *politus*, most asymmetries appear to be external, affecting the atrium (A) and Co in females (Fig 7, S1), and C and T in males (Figs 5 and 6 and S2). Genital parts involved in storage (Sd, S and Sa and glands), transport (Cd, Fd) and transfer of sperm (e) do not seem to be as modified. This scenario could be explained via mechanical fit and/or selective cooperation of the female [103, 104]. Here, the female genitalia grooves, for instance, the atrium (Fig 7a and 7d) anchor and control the coupling of the male palps conductor (Fig 5c and 5f). Directional asymmetry observed in pholcidae and oonopidae appear to be more related to the size and shape of sperm transfer and storage structures suggesting a functional specialization of one side over the other.

Besides the simple mechanical fit of genitalia, stimulatory cues may also be a driving factor in the evolution of DA. Spider genitalia were thought to be numb mechanical structures without nervous input. However, recent studies have found neurons in spider genitalia [112, 113] that might provide sensory input and stimulation during copulation. Similar asymmetries in shape and size have been found in males of some sepsid flies. Here, the asymmetric intromittent structures are rhythmically used to stimulate the female during copulation [114]. This hypothesis was not tested in the present work; however, the appearance of asymmetrycal sclerites (as seen in *T*. *politus*, *Metagonia* [17] and *Panjanje* [11]) and might be related to a differential stimulation of the female genitalia.

Changes in mating position have also been associated with many cases of DA in insect genitalia [1, 4, 12]. Unfortunately we were not able to test this in the case of *T*. *politus* using live specimens; nevertheless, observations in *Agroeca* [115] and other RTA spiders [13, 116] suggest that copulation is achieved by the male climbing over the female and stretching over a side while the female slightly turns her abdomen; this process is alternated between right and left side. In *T*. *politus*, female genital opening location makes it virtually impossible to have successful mating attempt from a right-side position. Instead, a male must insert both pedipalps always from the left side in relation to the female body. Morphological modifications like the difference in conductor shape (Figs 5c, 5f, 6b and 6c) and seemingly flatter tegulum of the left side (Fig 5e and 5f) are consistent with this hypothesis. In addition, this evidence seems to back the hypothesis discussed by Schilthuizen [3] and Huber, *et al*. [1] stating that in spiders asymmetry is most likely female-initiated and male changes appear as an evolutionary response.

## Conclusions

Genital evolution is a complex and interesting topic. The appearance of asymmetric morphologies is a puzzling phenomenon that has often been overlooked. Here we reported *T*. *politus* as the first case of directional asymmetry, and the first developmental asymmetry in male genitals, in Entelegynae spiders. We also searched for as many cases as possible in taxonomic literature; however, many more might be waiting to be (re)discovered. Our review revealed multiple origins of genital asymmetry in at least thirteen families, and in some cases (e.g. Oonopidae, Pholcidae, Theridiidae, Liocranidae) two or more within the same family. A correct assessment of genital asymmetry based on taxonomic legacy literature is difficult mainly due to the lack of data, description and illustration biases, and limited number of specimens and variation in descriptions.

As noted previously for genital asymmetry in insects and spiders, there is no single explanation for the evolution of this trait, but some generalizations can be made. In contrast to insects and other arthropod groups, the low number of genital asymmetric species in spiders might indicate that the appearance of these morphological modifications reduce subsequent speciation rates or even increase extinction rates; specialized lineages tend to have a reduced capacity to diversify and therefore might be considered evolutionary dead ends [117]. However, our observations indicate that cases of sexual asymmetry in spiders, although rare, are more common than was previously thought. Furthermore, they have evolved independently several times but rarely appear isolated and most of the times seem to be clustered within a genus or closely related genera, as in the cases of Oonopidae, Pholcidae, Theridiidae, and probably Liocranidae. The evolution of genital asymmetries in spiders might be a good candidate to be tested as a potential evolutionary dead end.

Several hypotheses for the appearance of asymmetry in spiders have been proposed and include natural selection [9, 102], sexual selection [11, 17] and antagonistic co-evolution [1, 15, 56] (not mutually exclusive). We considered *Echinotheridion* and *Tidarren* to be examples of antagonistic co-evolution where the male has evolved self-emasculation in response to the extreme sexual dimorphism in size and aggressive behavior in the female. No selection between left and right is apparent in these genera, thus no directionality is observed. DA cases like *T*. *politus* seem to support the hypothesis that correlates changes in mating position to genital asymmetry; however, other examples still need to be studied. DA in *T*. *politus* and some pholcid examples, AS in *Scotinella* and *Asygyna*, and CA cases in *Jacaena*, *Cithaeron* and *Trachelas* support the hypothesis of female-initiated asymmetry in spiders. However, male DA in Oonopidae and AS in some theridiids conflict with this explanation. Further and more detailed study on internal genitalia and comparative study of male right and left pedipalps may yield new and valuable information to explain the evolutionary pattern of genital asymmetry. We hope that this review will aid in the study, development and testing of hypotheses on sexual evolution. We specifically hope it sparks discussions on the complex interactions between males and females, and appearance of interesting phenomena like genital asymmetry.

## Supporting information

S1 Data Intra-specific variation female external genitalia. Standard views of sexual structures used to aid in DA comparison. One comparative plates of the epigyna ventral view is given. Scalebars = 0.5 mm. Individual pictures of five female specimens are also included.
(ZIP)

S2 Data Intra-specific variation of male genitalia. Standard views of sexual structures used to aid in DA comparison. Three comparative plates of the pedipalp, prolateral, retrolateral and ventral views are given. Top row = left pedipal; bottom row = right pedipal. Scalebars = 0.5 mm. Individual pictures of both palps from five specimens are also included.
(ZIP)
