# Supplementary figures and images for: Imperfect and askew: A review of asymmetric genitalia in araneomorph spiders (Araneae: Araneomorphae)
Source: PLoS One. 2020 Jun 15;15(6):e0220354. doi: 10.1371/journal.pone.0220354 (PMC7295216; doi:10.1371/journal.pone.0220354)

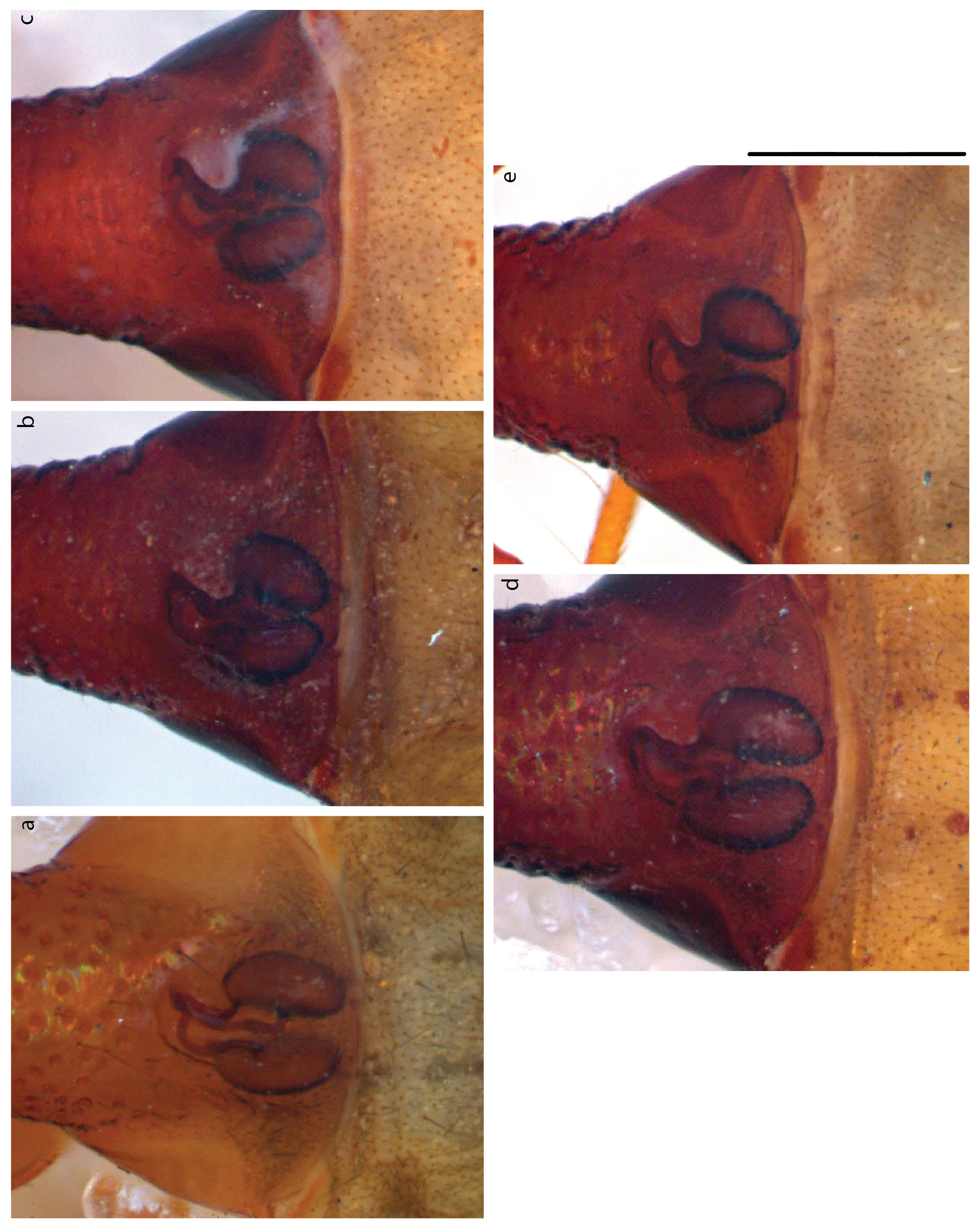

Supplement: S1 Data — Standard views of sexual structures used to aid in DA comparison. One comparative plates of the epigyna ventral view is given. Scalebars = 0.5 mm. Individual pictures of five female specimens are also included. (ZIP) [file pone.0220354.s001.zip › S1_Comp_pic_female/Asym_gen_COMP_EPI.jpg]

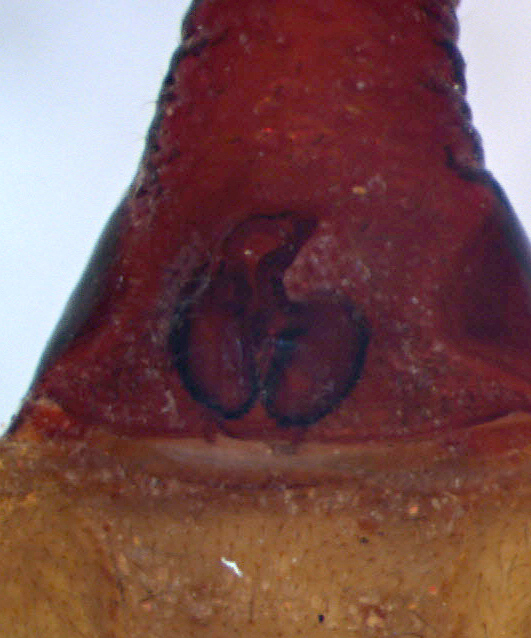

Supplement: S1 Data — Standard views of sexual structures used to aid in DA comparison. One comparative plates of the epigyna ventral view is given. Scalebars = 0.5 mm. Individual pictures of five female specimens are also included. (ZIP) [file pone.0220354.s001.zip › S1_Comp_pic_female/Female/Teuta_polit_fp_epiv_06_RMNH.5084632.JPG]

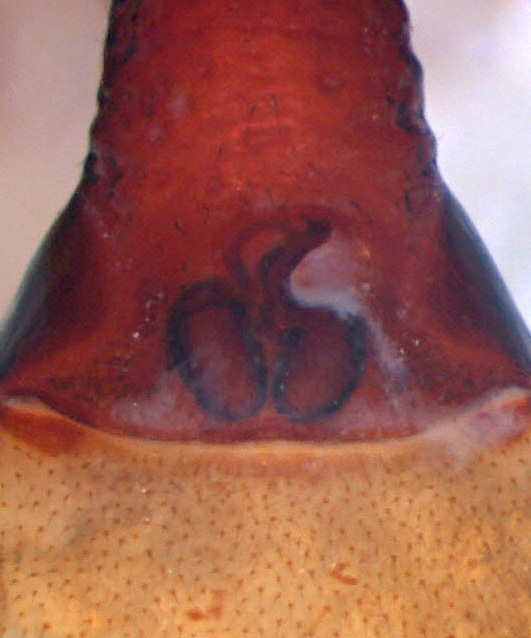

Supplement: S1 Data — Standard views of sexual structures used to aid in DA comparison. One comparative plates of the epigyna ventral view is given. Scalebars = 0.5 mm. Individual pictures of five female specimens are also included. (ZIP) [file pone.0220354.s001.zip › S1_Comp_pic_female/Female/Teuta_polit_fp_epiv_07_RMNH.5084632.JPG]

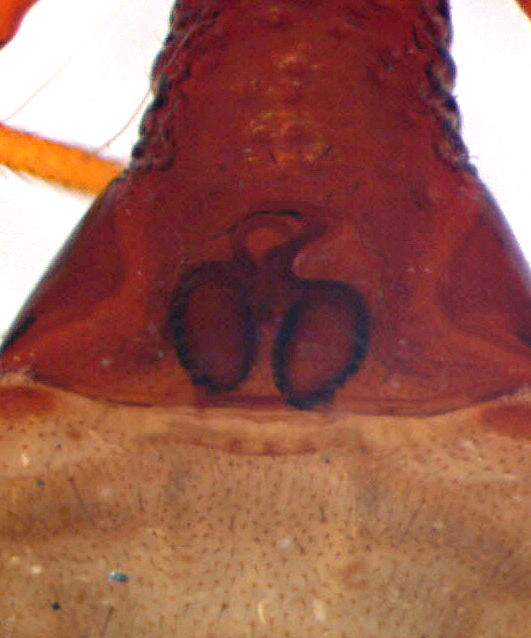

Supplement: S1 Data — Standard views of sexual structures used to aid in DA comparison. One comparative plates of the epigyna ventral view is given. Scalebars = 0.5 mm. Individual pictures of five female specimens are also included. (ZIP) [file pone.0220354.s001.zip › S1_Comp_pic_female/Female/Teuta_polit_fp_epiv_08_RMNH.5084641.JPG]

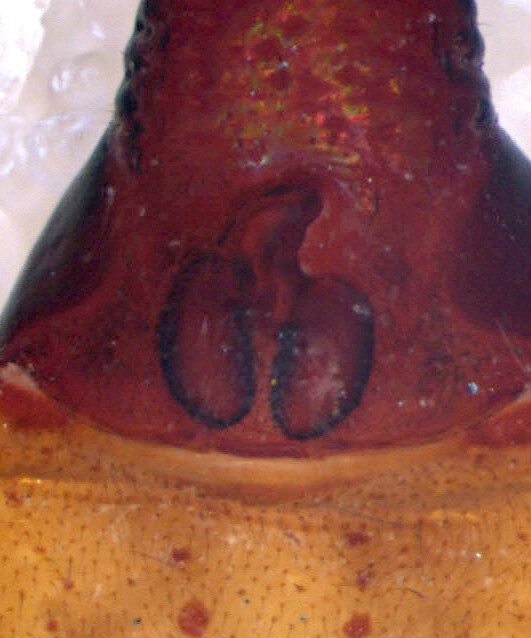

Supplement: S1 Data — Standard views of sexual structures used to aid in DA comparison. One comparative plates of the epigyna ventral view is given. Scalebars = 0.5 mm. Individual pictures of five female specimens are also included. (ZIP) [file pone.0220354.s001.zip › S1_Comp_pic_female/Female/Teuta_polit_fp_epiv_09_RMNH.5084647.JPG]

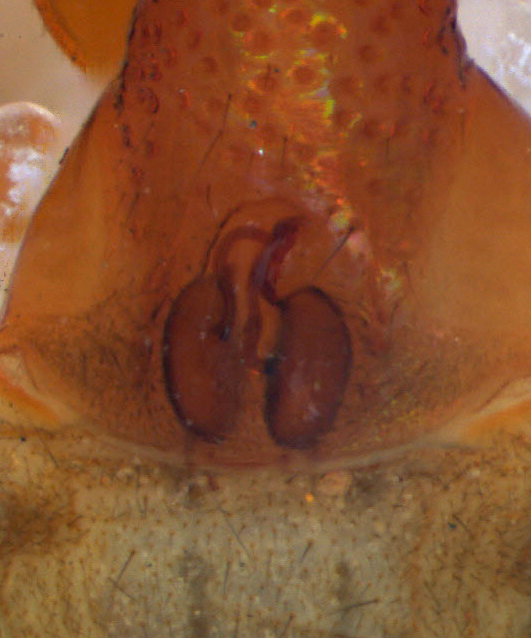

Supplement: S1 Data — Standard views of sexual structures used to aid in DA comparison. One comparative plates of the epigyna ventral view is given. Scalebars = 0.5 mm. Individual pictures of five female specimens are also included. (ZIP) [file pone.0220354.s001.zip › S1_Comp_pic_female/Female/Teuta_polit_fp_epiv_10_RMNH.5084647.JPG]

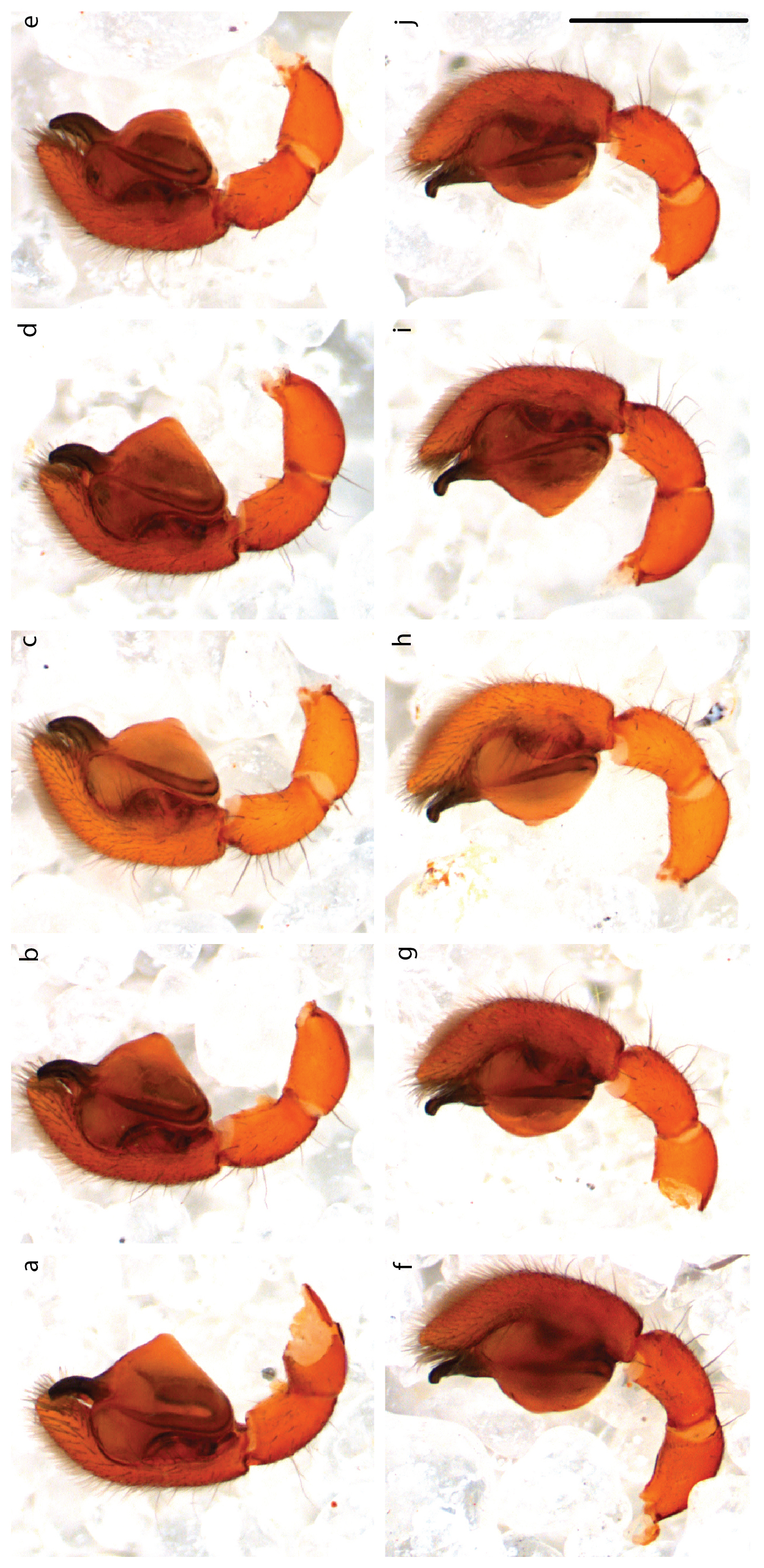

Supplement: S2 Data — Standard views of sexual structures used to aid in DA comparison. Three comparative plates of the pedipalp, prolateral, retrolateral and ventral views are given. Top row = left pedipal; bottom row = right pedipal. Scalebars = 0.5 mm. Individual pictures of both palps from five specimens are also included. (ZIP) [file pone.0220354.s002.zip › S2_Comp_pic_male/Asym_gen_COMP_palp.jpg]

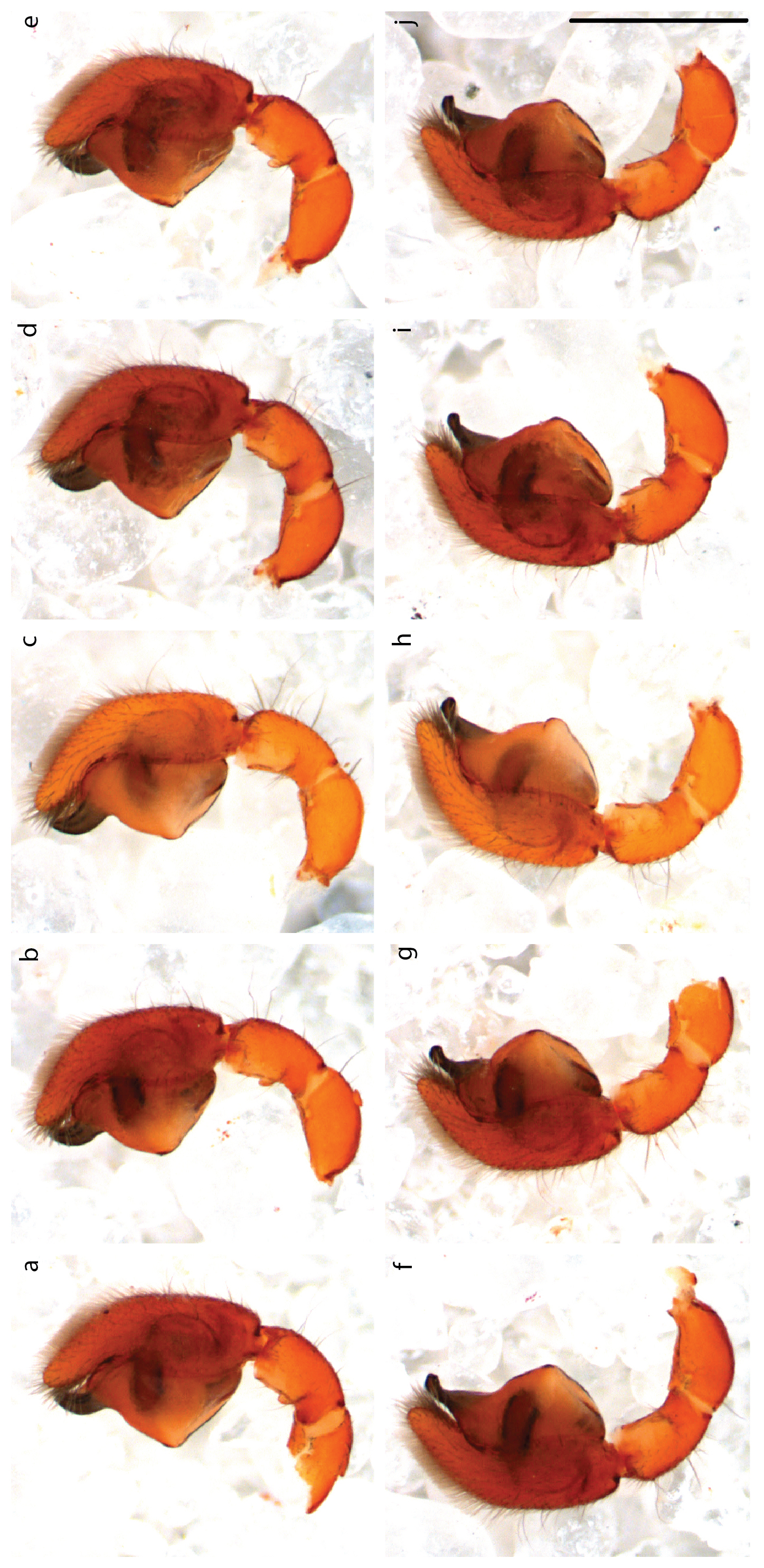

Supplement: S2 Data — Standard views of sexual structures used to aid in DA comparison. Three comparative plates of the pedipalp, prolateral, retrolateral and ventral views are given. Top row = left pedipal; bottom row = right pedipal. Scalebars = 0.5 mm. Individual pictures of both palps from five specimens are also included. (ZIP) [file pone.0220354.s002.zip › S2_Comp_pic_male/Asym_gen_COMP_palr.jpg]

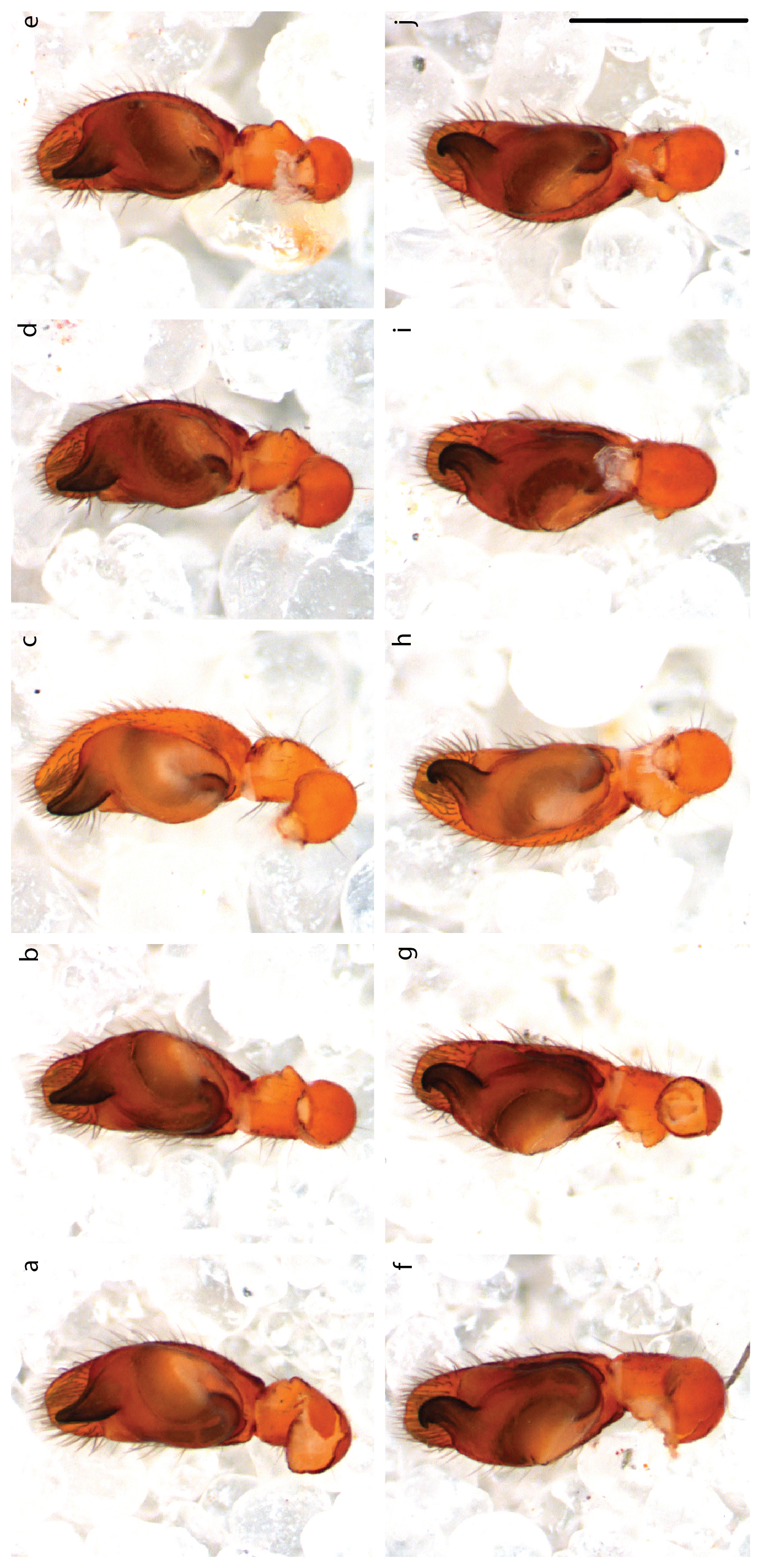

Supplement: S2 Data — Standard views of sexual structures used to aid in DA comparison. Three comparative plates of the pedipalp, prolateral, retrolateral and ventral views are given. Top row = left pedipal; bottom row = right pedipal. Scalebars = 0.5 mm. Individual pictures of both palps from five specimens are also included. (ZIP) [file pone.0220354.s002.zip › S2_Comp_pic_male/Asym_gen_COMP_palV.jpg]

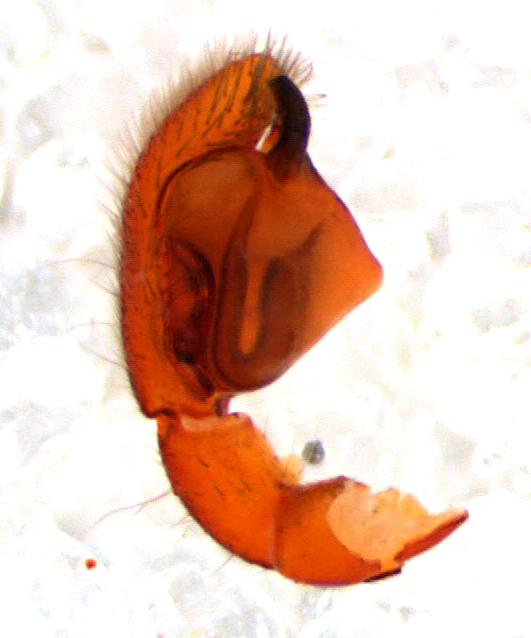

Supplement: S2 Data — Standard views of sexual structures used to aid in DA comparison. Three comparative plates of the pedipalp, prolateral, retrolateral and ventral views are given. Top row = left pedipal; bottom row = right pedipal. Scalebars = 0.5 mm. Individual pictures of both palps from five specimens are also included. (ZIP) [file pone.0220354.s002.zip › S2_Comp_pic_male/Left/Teuta_polit_mp_palp_L_01_RMNH.5084632.JPG]

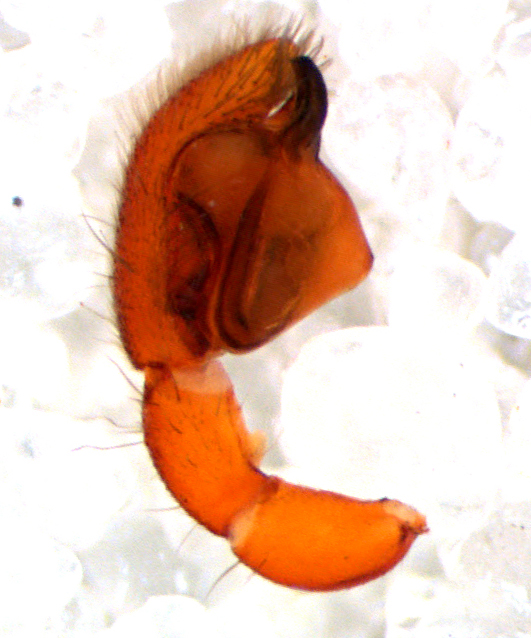

Supplement: S2 Data — Standard views of sexual structures used to aid in DA comparison. Three comparative plates of the pedipalp, prolateral, retrolateral and ventral views are given. Top row = left pedipal; bottom row = right pedipal. Scalebars = 0.5 mm. Individual pictures of both palps from five specimens are also included. (ZIP) [file pone.0220354.s002.zip › S2_Comp_pic_male/Left/Teuta_polit_mp_palp_L_02_RMNH.5084632.JPG]

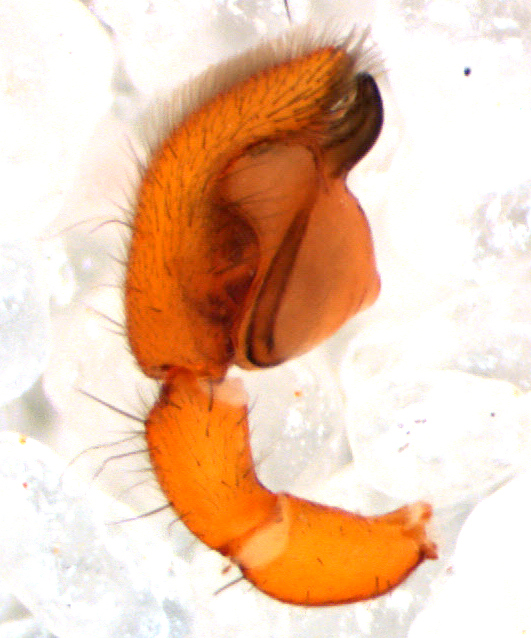

Supplement: S2 Data — Standard views of sexual structures used to aid in DA comparison. Three comparative plates of the pedipalp, prolateral, retrolateral and ventral views are given. Top row = left pedipal; bottom row = right pedipal. Scalebars = 0.5 mm. Individual pictures of both palps from five specimens are also included. (ZIP) [file pone.0220354.s002.zip › S2_Comp_pic_male/Left/Teuta_polit_mp_palp_L_03_RMNH.5084632.JPG]

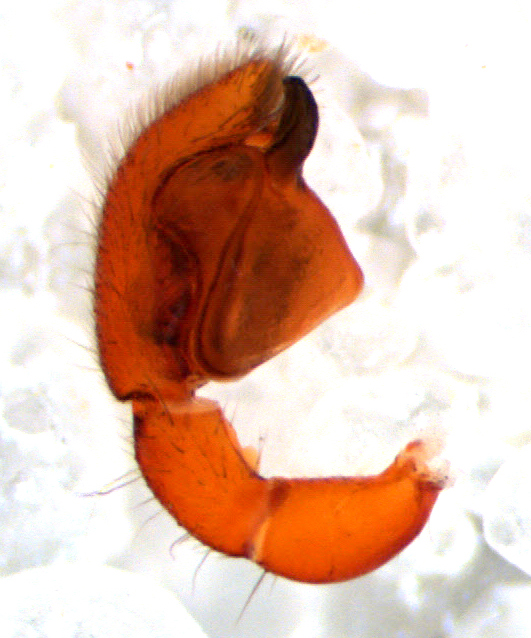

Supplement: S2 Data — Standard views of sexual structures used to aid in DA comparison. Three comparative plates of the pedipalp, prolateral, retrolateral and ventral views are given. Top row = left pedipal; bottom row = right pedipal. Scalebars = 0.5 mm. Individual pictures of both palps from five specimens are also included. (ZIP) [file pone.0220354.s002.zip › S2_Comp_pic_male/Left/Teuta_polit_mp_palp_L_04_RMNH.5084632.JPG]

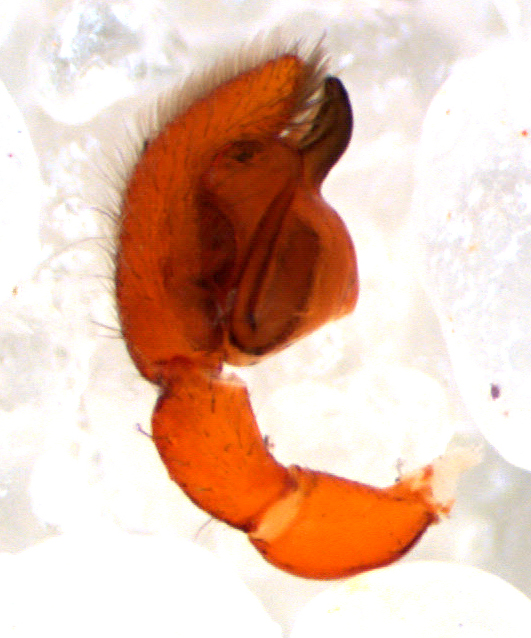

Supplement: S2 Data — Standard views of sexual structures used to aid in DA comparison. Three comparative plates of the pedipalp, prolateral, retrolateral and ventral views are given. Top row = left pedipal; bottom row = right pedipal. Scalebars = 0.5 mm. Individual pictures of both palps from five specimens are also included. (ZIP) [file pone.0220354.s002.zip › S2_Comp_pic_male/Left/Teuta_polit_mp_palp_L_05_sRMNH.5084632.JPG]

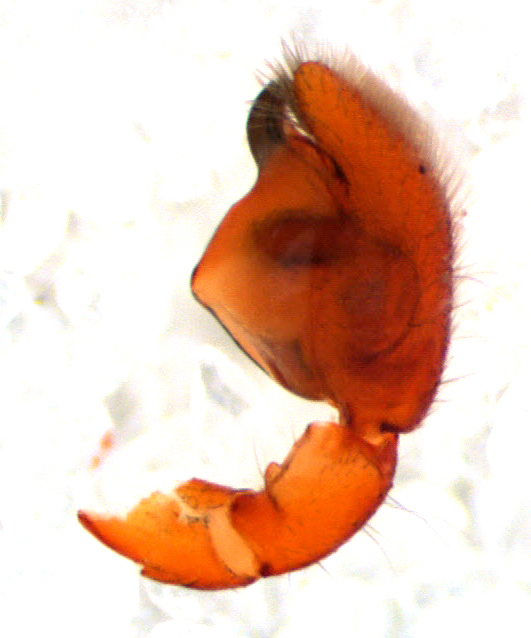

Supplement: S2 Data — Standard views of sexual structures used to aid in DA comparison. Three comparative plates of the pedipalp, prolateral, retrolateral and ventral views are given. Top row = left pedipal; bottom row = right pedipal. Scalebars = 0.5 mm. Individual pictures of both palps from five specimens are also included. (ZIP) [file pone.0220354.s002.zip › S2_Comp_pic_male/Left/Teuta_polit_mp_palr_L_01_RMNH.5084632.JPG]

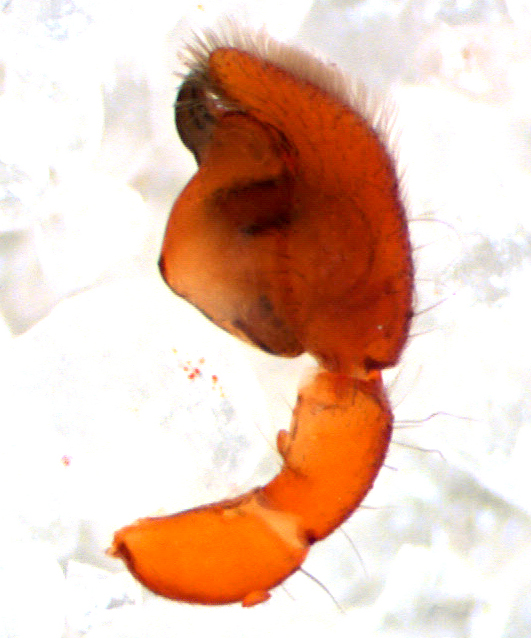

Supplement: S2 Data — Standard views of sexual structures used to aid in DA comparison. Three comparative plates of the pedipalp, prolateral, retrolateral and ventral views are given. Top row = left pedipal; bottom row = right pedipal. Scalebars = 0.5 mm. Individual pictures of both palps from five specimens are also included. (ZIP) [file pone.0220354.s002.zip › S2_Comp_pic_male/Left/Teuta_polit_mp_palr_L_02_RMNH.5084632.JPG]

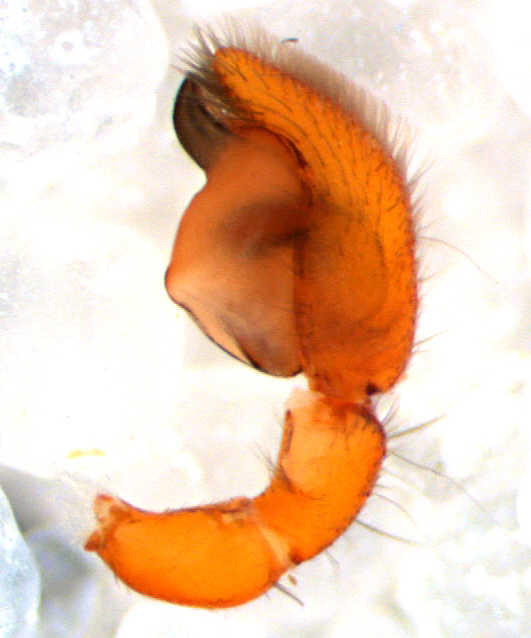

Supplement: S2 Data — Standard views of sexual structures used to aid in DA comparison. Three comparative plates of the pedipalp, prolateral, retrolateral and ventral views are given. Top row = left pedipal; bottom row = right pedipal. Scalebars = 0.5 mm. Individual pictures of both palps from five specimens are also included. (ZIP) [file pone.0220354.s002.zip › S2_Comp_pic_male/Left/Teuta_polit_mp_palr_L_03_RMNH.5084632.JPG]

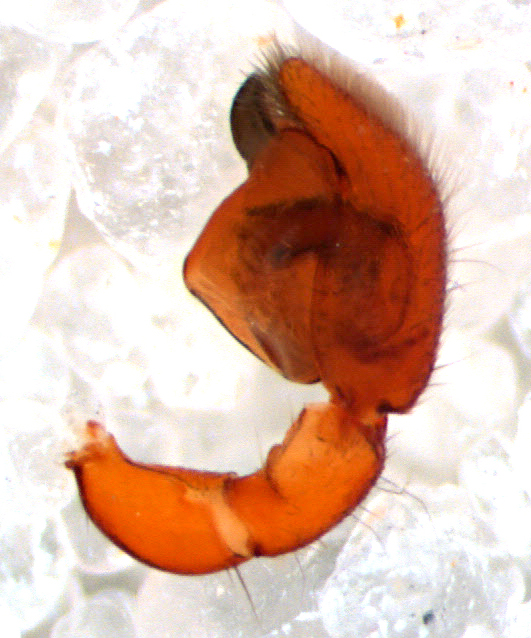

Supplement: S2 Data — Standard views of sexual structures used to aid in DA comparison. Three comparative plates of the pedipalp, prolateral, retrolateral and ventral views are given. Top row = left pedipal; bottom row = right pedipal. Scalebars = 0.5 mm. Individual pictures of both palps from five specimens are also included. (ZIP) [file pone.0220354.s002.zip › S2_Comp_pic_male/Left/Teuta_polit_mp_palr_L_04_RMNH.5084632.JPG]

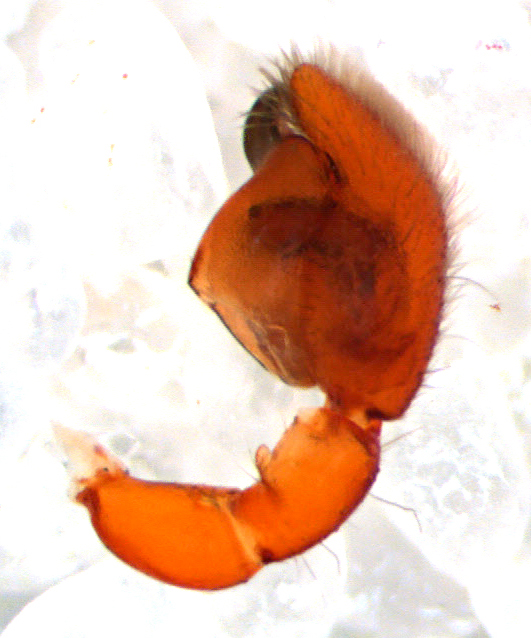

Supplement: S2 Data — Standard views of sexual structures used to aid in DA comparison. Three comparative plates of the pedipalp, prolateral, retrolateral and ventral views are given. Top row = left pedipal; bottom row = right pedipal. Scalebars = 0.5 mm. Individual pictures of both palps from five specimens are also included. (ZIP) [file pone.0220354.s002.zip › S2_Comp_pic_male/Left/Teuta_polit_mp_palr_L_05_RMNH.5084632.JPG]

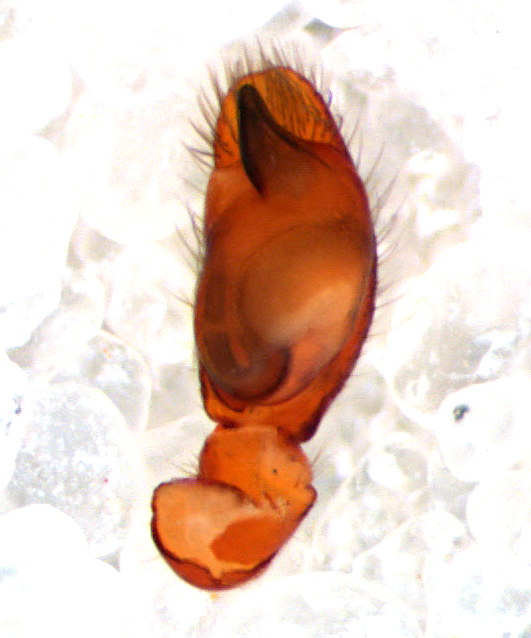

Supplement: S2 Data — Standard views of sexual structures used to aid in DA comparison. Three comparative plates of the pedipalp, prolateral, retrolateral and ventral views are given. Top row = left pedipal; bottom row = right pedipal. Scalebars = 0.5 mm. Individual pictures of both palps from five specimens are also included. (ZIP) [file pone.0220354.s002.zip › S2_Comp_pic_male/Left/Teuta_polit_mp_palv_L_01_RMNH.5084632.JPG]

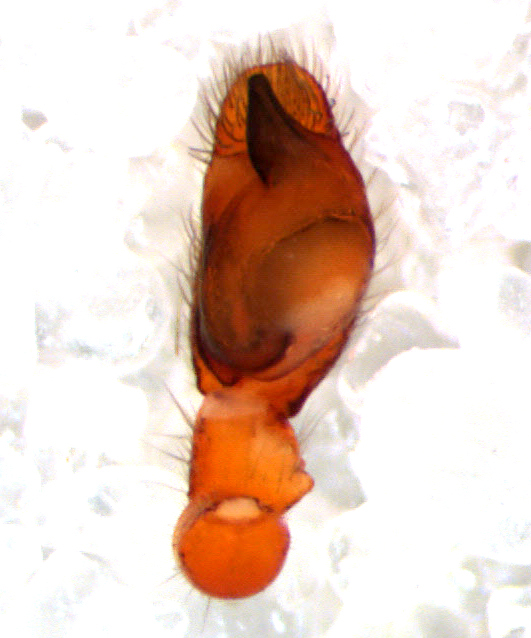

Supplement: S2 Data — Standard views of sexual structures used to aid in DA comparison. Three comparative plates of the pedipalp, prolateral, retrolateral and ventral views are given. Top row = left pedipal; bottom row = right pedipal. Scalebars = 0.5 mm. Individual pictures of both palps from five specimens are also included. (ZIP) [file pone.0220354.s002.zip › S2_Comp_pic_male/Left/Teuta_polit_mp_palv_L_02_RMNH.5084632.JPG]

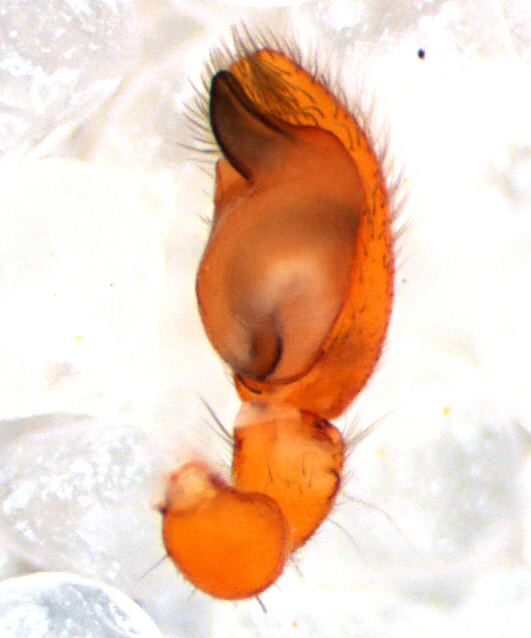

Supplement: S2 Data — Standard views of sexual structures used to aid in DA comparison. Three comparative plates of the pedipalp, prolateral, retrolateral and ventral views are given. Top row = left pedipal; bottom row = right pedipal. Scalebars = 0.5 mm. Individual pictures of both palps from five specimens are also included. (ZIP) [file pone.0220354.s002.zip › S2_Comp_pic_male/Left/Teuta_polit_mp_palv_L_03_RMNH.5084632.JPG]

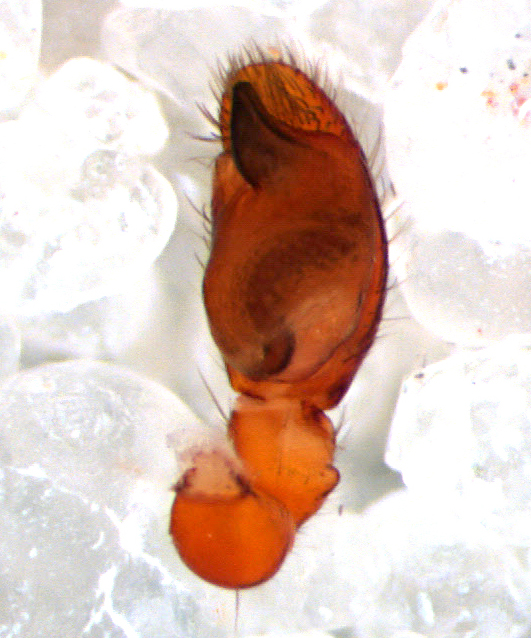

Supplement: S2 Data — Standard views of sexual structures used to aid in DA comparison. Three comparative plates of the pedipalp, prolateral, retrolateral and ventral views are given. Top row = left pedipal; bottom row = right pedipal. Scalebars = 0.5 mm. Individual pictures of both palps from five specimens are also included. (ZIP) [file pone.0220354.s002.zip › S2_Comp_pic_male/Left/Teuta_polit_mp_palv_L_04_RMNH.5084632.JPG]

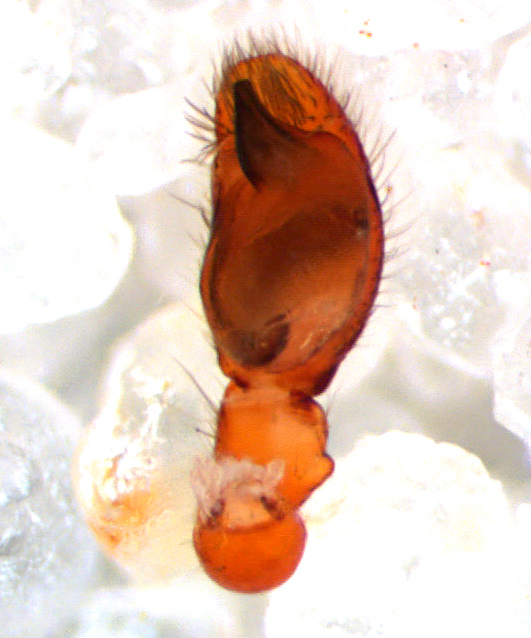

Supplement: S2 Data — Standard views of sexual structures used to aid in DA comparison. Three comparative plates of the pedipalp, prolateral, retrolateral and ventral views are given. Top row = left pedipal; bottom row = right pedipal. Scalebars = 0.5 mm. Individual pictures of both palps from five specimens are also included. (ZIP) [file pone.0220354.s002.zip › S2_Comp_pic_male/Left/Teuta_polit_mp_palv_L_05_RMNH.5084632.JPG]

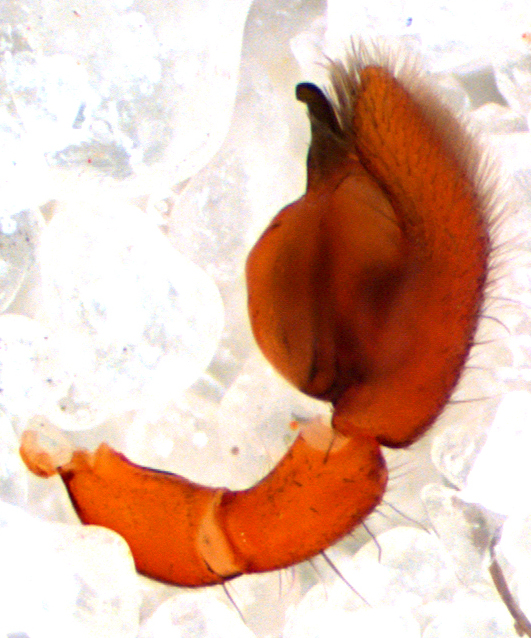

Supplement: S2 Data — Standard views of sexual structures used to aid in DA comparison. Three comparative plates of the pedipalp, prolateral, retrolateral and ventral views are given. Top row = left pedipal; bottom row = right pedipal. Scalebars = 0.5 mm. Individual pictures of both palps from five specimens are also included. (ZIP) [file pone.0220354.s002.zip › S2_Comp_pic_male/Right/Teuta_polit_mp_palp_R_01_RMNH.5084632.jpg]

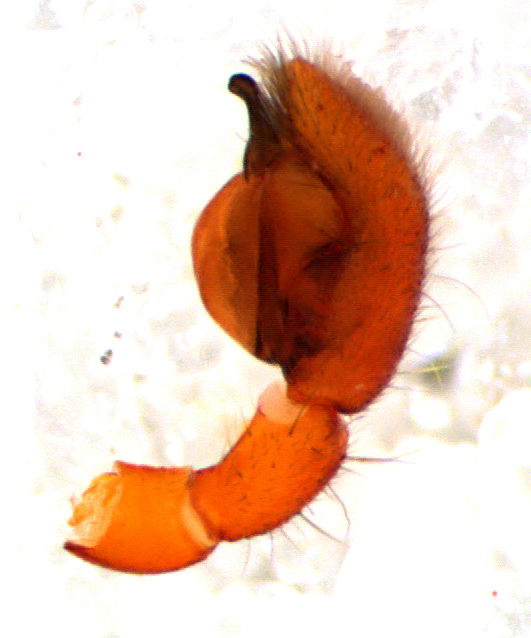

Supplement: S2 Data — Standard views of sexual structures used to aid in DA comparison. Three comparative plates of the pedipalp, prolateral, retrolateral and ventral views are given. Top row = left pedipal; bottom row = right pedipal. Scalebars = 0.5 mm. Individual pictures of both palps from five specimens are also included. (ZIP) [file pone.0220354.s002.zip › S2_Comp_pic_male/Right/Teuta_polit_mp_palp_R_02_RMNH.5084632.JPG]

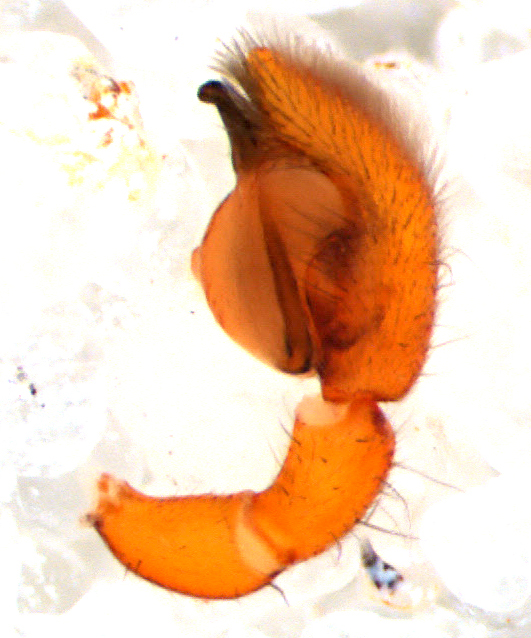

Supplement: S2 Data — Standard views of sexual structures used to aid in DA comparison. Three comparative plates of the pedipalp, prolateral, retrolateral and ventral views are given. Top row = left pedipal; bottom row = right pedipal. Scalebars = 0.5 mm. Individual pictures of both palps from five specimens are also included. (ZIP) [file pone.0220354.s002.zip › S2_Comp_pic_male/Right/Teuta_polit_mp_palp_R_03_RMNH.5084632.JPG]

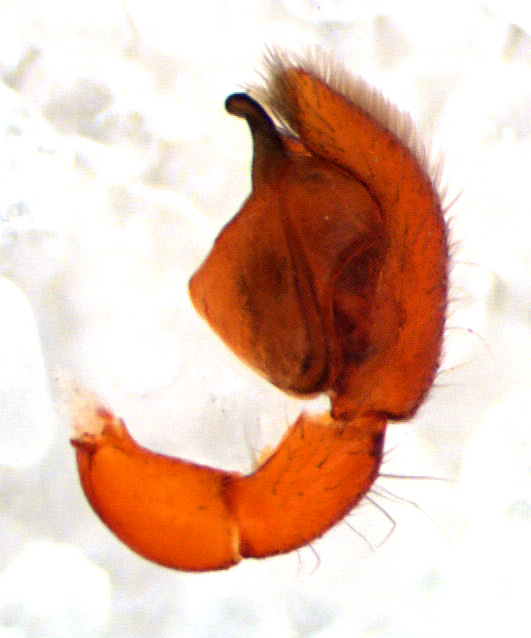

Supplement: S2 Data — Standard views of sexual structures used to aid in DA comparison. Three comparative plates of the pedipalp, prolateral, retrolateral and ventral views are given. Top row = left pedipal; bottom row = right pedipal. Scalebars = 0.5 mm. Individual pictures of both palps from five specimens are also included. (ZIP) [file pone.0220354.s002.zip › S2_Comp_pic_male/Right/Teuta_polit_mp_palp_R_04_RMNH.5084632.JPG]

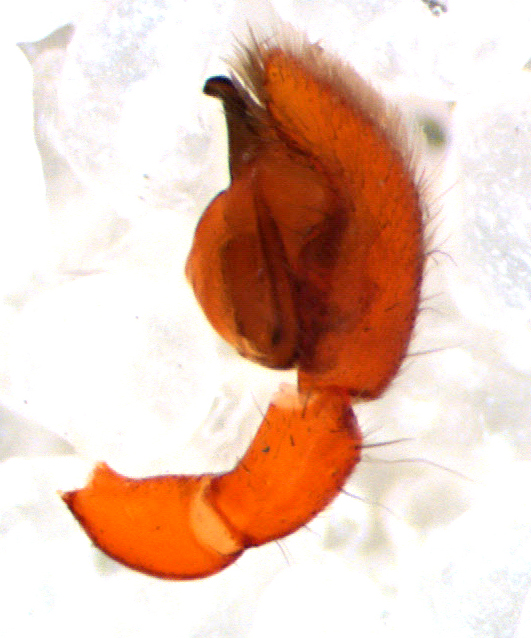

Supplement: S2 Data — Standard views of sexual structures used to aid in DA comparison. Three comparative plates of the pedipalp, prolateral, retrolateral and ventral views are given. Top row = left pedipal; bottom row = right pedipal. Scalebars = 0.5 mm. Individual pictures of both palps from five specimens are also included. (ZIP) [file pone.0220354.s002.zip › S2_Comp_pic_male/Right/Teuta_polit_mp_palp_R_05_RMNH.5084632.JPG]

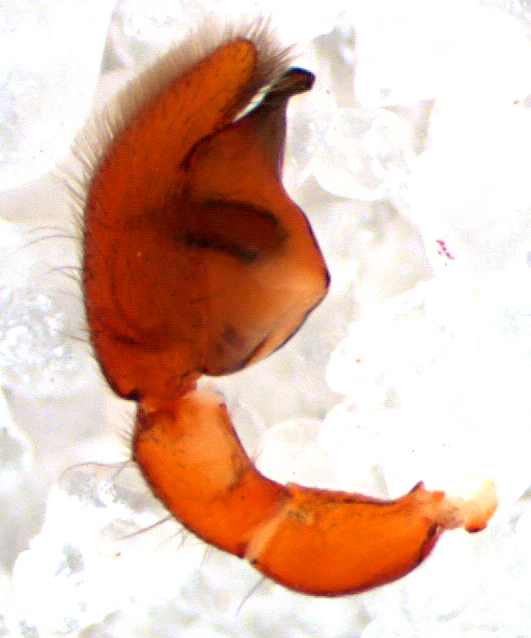

Supplement: S2 Data — Standard views of sexual structures used to aid in DA comparison. Three comparative plates of the pedipalp, prolateral, retrolateral and ventral views are given. Top row = left pedipal; bottom row = right pedipal. Scalebars = 0.5 mm. Individual pictures of both palps from five specimens are also included. (ZIP) [file pone.0220354.s002.zip › S2_Comp_pic_male/Right/Teuta_polit_mp_palr_R_01_RMNH.5084632.JPG]

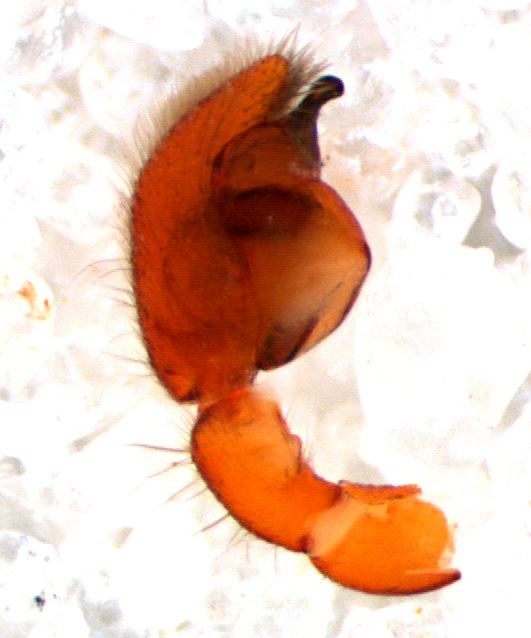

Supplement: S2 Data — Standard views of sexual structures used to aid in DA comparison. Three comparative plates of the pedipalp, prolateral, retrolateral and ventral views are given. Top row = left pedipal; bottom row = right pedipal. Scalebars = 0.5 mm. Individual pictures of both palps from five specimens are also included. (ZIP) [file pone.0220354.s002.zip › S2_Comp_pic_male/Right/Teuta_polit_mp_palr_R_02_RMNH.5084632.JPG]

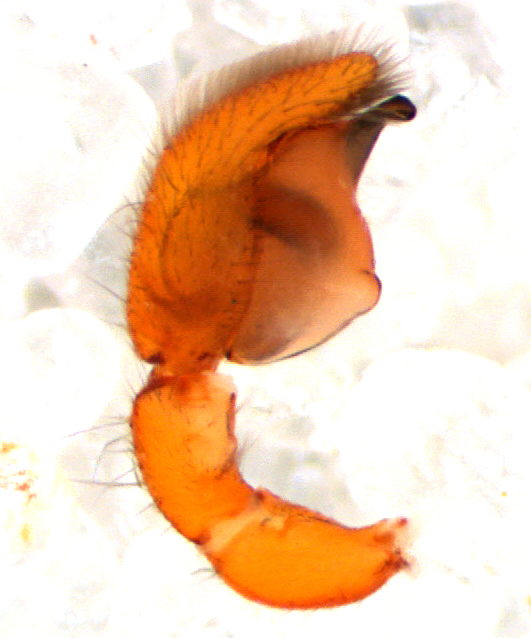

Supplement: S2 Data — Standard views of sexual structures used to aid in DA comparison. Three comparative plates of the pedipalp, prolateral, retrolateral and ventral views are given. Top row = left pedipal; bottom row = right pedipal. Scalebars = 0.5 mm. Individual pictures of both palps from five specimens are also included. (ZIP) [file pone.0220354.s002.zip › S2_Comp_pic_male/Right/Teuta_polit_mp_palr_R_03_RMNH.5084632.JPG]

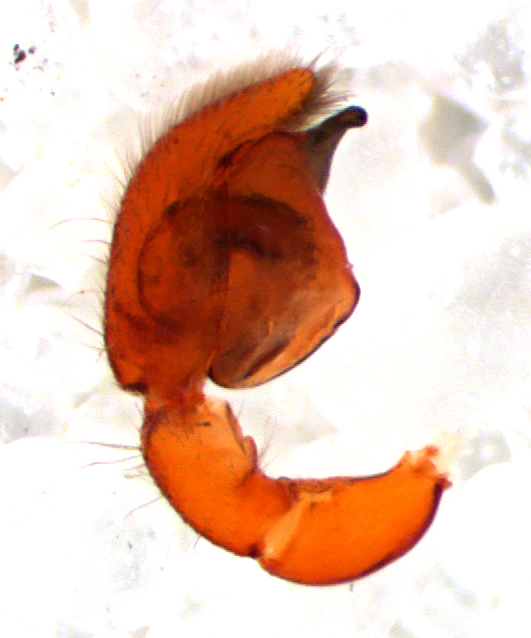

Supplement: S2 Data — Standard views of sexual structures used to aid in DA comparison. Three comparative plates of the pedipalp, prolateral, retrolateral and ventral views are given. Top row = left pedipal; bottom row = right pedipal. Scalebars = 0.5 mm. Individual pictures of both palps from five specimens are also included. (ZIP) [file pone.0220354.s002.zip › S2_Comp_pic_male/Right/Teuta_polit_mp_palr_R_04_RMNH.5084632.JPG]

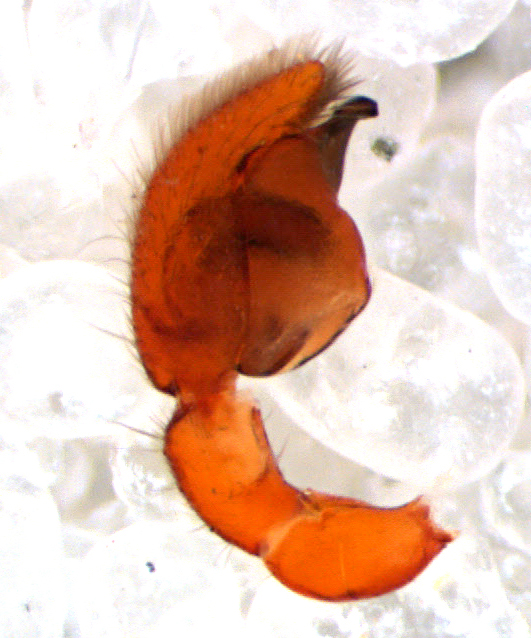

Supplement: S2 Data — Standard views of sexual structures used to aid in DA comparison. Three comparative plates of the pedipalp, prolateral, retrolateral and ventral views are given. Top row = left pedipal; bottom row = right pedipal. Scalebars = 0.5 mm. Individual pictures of both palps from five specimens are also included. (ZIP) [file pone.0220354.s002.zip › S2_Comp_pic_male/Right/Teuta_polit_mp_palr_R_05_RMNH.5084632.JPG]

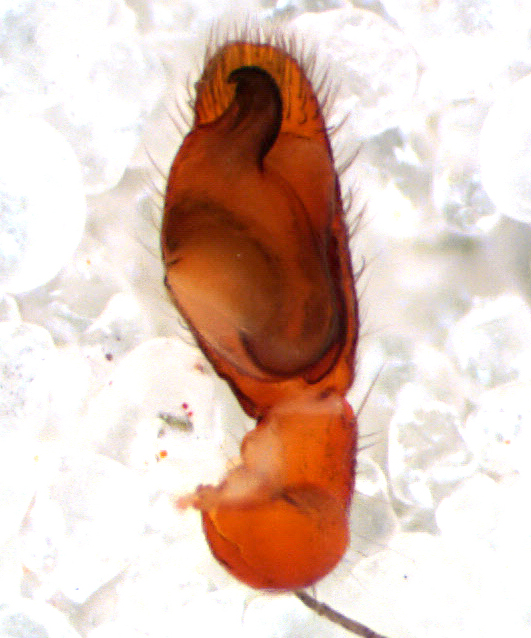

Supplement: S2 Data — Standard views of sexual structures used to aid in DA comparison. Three comparative plates of the pedipalp, prolateral, retrolateral and ventral views are given. Top row = left pedipal; bottom row = right pedipal. Scalebars = 0.5 mm. Individual pictures of both palps from five specimens are also included. (ZIP) [file pone.0220354.s002.zip › S2_Comp_pic_male/Right/Teuta_polit_mp_palv_R_01_RMNH.5084632.JPG]

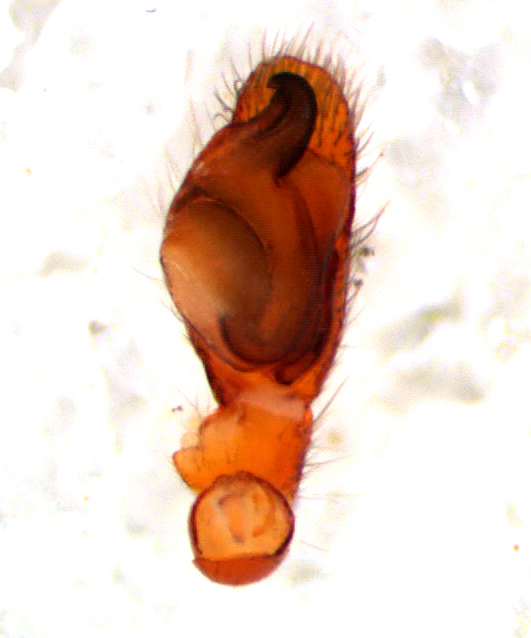

Supplement: S2 Data — Standard views of sexual structures used to aid in DA comparison. Three comparative plates of the pedipalp, prolateral, retrolateral and ventral views are given. Top row = left pedipal; bottom row = right pedipal. Scalebars = 0.5 mm. Individual pictures of both palps from five specimens are also included. (ZIP) [file pone.0220354.s002.zip › S2_Comp_pic_male/Right/Teuta_polit_mp_palv_R_02_RMNH.5084632.JPG]

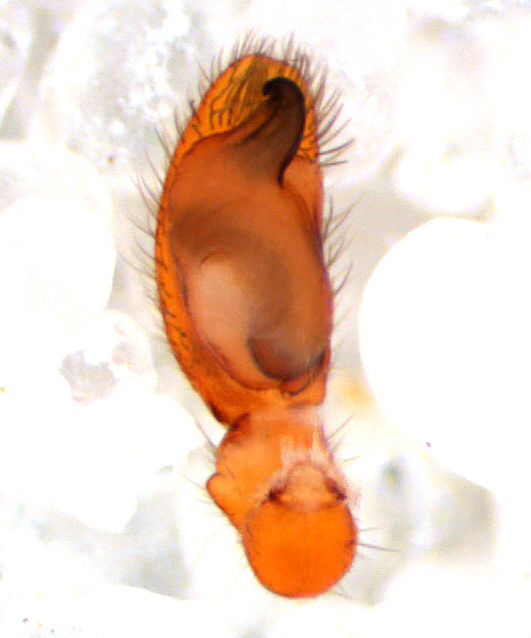

Supplement: S2 Data — Standard views of sexual structures used to aid in DA comparison. Three comparative plates of the pedipalp, prolateral, retrolateral and ventral views are given. Top row = left pedipal; bottom row = right pedipal. Scalebars = 0.5 mm. Individual pictures of both palps from five specimens are also included. (ZIP) [file pone.0220354.s002.zip › S2_Comp_pic_male/Right/Teuta_polit_mp_palv_R_03_RMNH.5084632.JPG]

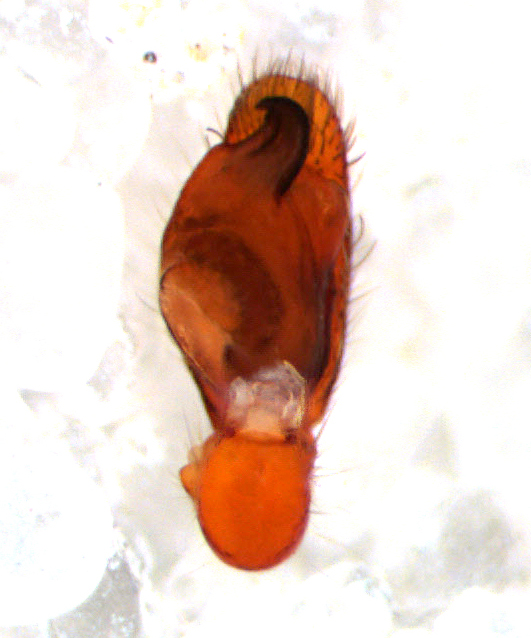

Supplement: S2 Data — Standard views of sexual structures used to aid in DA comparison. Three comparative plates of the pedipalp, prolateral, retrolateral and ventral views are given. Top row = left pedipal; bottom row = right pedipal. Scalebars = 0.5 mm. Individual pictures of both palps from five specimens are also included. (ZIP) [file pone.0220354.s002.zip › S2_Comp_pic_male/Right/Teuta_polit_mp_palv_R_04_RMNH.5084632.JPG]

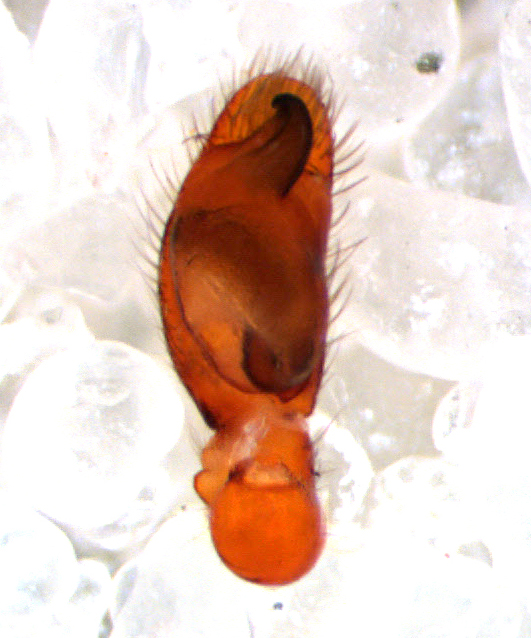

Supplement: S2 Data — Standard views of sexual structures used to aid in DA comparison. Three comparative plates of the pedipalp, prolateral, retrolateral and ventral views are given. Top row = left pedipal; bottom row = right pedipal. Scalebars = 0.5 mm. Individual pictures of both palps from five specimens are also included. (ZIP) [file pone.0220354.s002.zip › S2_Comp_pic_male/Right/Teuta_polit_mp_palv_R_05_RMNH.5084632.JPG]
